# Tubulointerstitial nephritis antigen-like 1 from cancer-associated fibroblasts contribute to the progression of diffuse-type gastric cancers through the interaction with integrin β1

**DOI:** 10.1186/s12967-024-04963-9

**Published:** 2024-02-14

**Authors:** Dagyeong Lee, In-Hye Ham, Hye Jeong Oh, Dong Min Lee, Jung Hwan Yoon, Sang-Yong Son, Tae-Min Kim, Jae-Young Kim, Sang-Uk Han, Hoon Hur

**Affiliations:** 1 Department of Surgery, Ajou University School of Medicine, Suwon, Republic of Korea; 2 Cancer Biology Graduate Program, Ajou University School of Medicine Suwon, Suwon, Republic of Korea; 3 AI-Super Convergence KIURI Translational Research Center, Ajou University School of Medicine, Suwon, Republic of Korea; 4 Inflamm-Aging Translational Research Center, Ajou University School of Medicine, Suwon, Republic of Korea; 5 Department of Pathology, College of Medicine, The Catholic University of Korea, Seoul, Republic of Korea; 6 Functional RNomics Research Center, College of Medicine, The Catholic University of Korea Seoul, Seoul, Republic of Korea; 7 Department of Medical Informatics, College of Medicine, The Catholic University of Korea, Seoul, Republic of Korea; 8 Cancer Research Institute, College of Medicine, The Catholic University of Korea, Seoul, Republic of Korea; 9 Department of Biomedicine and Health Science, Graduate School, The Catholic University of Korea, Seoul, Republic of Korea; 10 Graduate School of Analytical Science and Technology (GRAST), Chungnam National University, Daejeon, Republic of Korea

**Keywords:** Diffuse-type gastric cancer, Tumor microenvironment, Cancer-associated fibroblasts, Tubulointerstitial nephritis antigen-like 1, Integrin beta 1

## Abstract

**Background:**

Tumor cells of diffuse-type gastric cancer (DGC) are discohesive and infiltrate into the stroma as single cells or small subgroups, so the stroma significantly impacts DGC progression. Cancer-associated fibroblasts (CAFs) are major components of the tumor stroma. Here, we identified CAF-specific secreted molecules and investigated the mechanism underlying CAF-induced DGC progression.

**Methods:**

We conducted transcriptome analysis for paired normal fibroblast (NF)-CAF isolated from DGC patient tissues and proteomics for conditioned media (CM) of fibroblasts. The effects of fibroblasts on cancer cells were examined by transwell migration and soft agar assays, western blotting, and in vivo. We confirmed the effect of blocking tubulointerstitial nephritis antigen-like 1 (TINAGL1) in CAFs using siRNA or shRNA. We evaluated the expression of TINAGL1 protein in frozen tissues of DGC and paired normal stomach and mRNA in formalin-fixed, paraffin-embedded (FFPE) tissue using RNA in-situ hybridization (RNA-ISH).

**Results:**

CAFs more highly expressed TINAGL1 than NFs. The co-culture of CAFs increased migration and tumorigenesis of DGC. Moreover, CAFs enhanced the phosphorylation of focal adhesion kinase (FAK) and mesenchymal marker expression in DGC cells. In an animal study, DGC tumors co-injected with CAFs showed aggressive phenotypes, including lymph node metastasis. However, increased phosphorylation of FAK and migration were reduced by blocking TINAGL1 in CAFs. In the tissues of DGC patients, TINAGL1 was higher in cancer than paired normal tissues and detected with collagen type I alpha 1 chain (COL1A1) in the same spot. Furthermore, high TINAGL1 expression was significantly correlated with poor prognosis in several public databases and our patient cohort diagnosed with DGC.

**Conclusions:**

These results indicate that TINAGL1 secreted by CAFs induces phosphorylation of FAK in DGC cells and promotes tumor progression. Thus, targeting TINAGL1 in CAFs can be a novel therapeutic strategy for DGC.

**Supplementary Information:**

The online version contains supplementary material available at 10.1186/s12967-024-04963-9.

## Background

Gastric cancer (GC) is the fourth leading cause of cancer-related deaths worldwide [1]. The incidence rates of GC are highest in Eastern Asia, including Korea, and more frequent in men than in women. Although the survival rates of GC have improved, the five-year survival rates of patients diagnosed at the distant metastatic stage, which account for approximately 10% of GC, are only 5.7–6.8% [2]. According to Lauren’s classification, GC is histologically categorized as intestinal- and diffuse-type gastric cancer [3]. In intestinal-type gastric cancer, cancer cells are well differentiated and form glands. On the other hand, cancer cells are poorly differentiated and do not form glands in DGC. DGC represents 40–50% of all GC cases, and patients with DGC have a worse prognosis compared to those suffering from other types of cancer [4, 5]. The main reasons for poor prognosis are earlier recurrence and prominent metastasis to the peritoneum [6]. This classification system has been used in clinics, but treatment guidelines for patients with GC have not taken these histological classifications into account [7, 8]. In DGC, cancer cells are discohesive and infiltrate the stroma as single cells or small subgroups without forming tubular glands [9]. In addition, the molecular classifications by the Asian Cancer Research Group revealed that DGC overlaps with the epithelial-to-mesenchymal transition (EMT) subtype, which presents with a higher metastatic potential and worse prognosis than other subtypes [10]. Consequently, the interactions between cancer cells and tumor stroma could be a crucial contributor to the metastasis and progression of DGC; therefore, these mechanisms should be clarified to identify specific targets for patients with DGC.

Profuse fibrotic stroma has been considered the major feature of aggressive malignant tumors such as pancreatic cancer, and it was a characteristic of DGC, in contrast to intestinal gastric cancer. Notably, increasing evidence suggests that enriched fibrotic stroma is significantly correlated with poor outcomes in GC [11, 12]. Our previous study also revealed that profuse fibrotic stroma in signet ring cell gastric carcinoma, which is mainly classified as DGC, was significantly correlated with poor prognosis in patients [13]. Cancer-associated fibroblasts (CAFs) are major components of fibrotic stroma, but CAFs have not been targeted in clinical settings. The origin of CAFs is not exactly defined, but the concept that CAFs are the result of normal fibroblast activation is generally accepted [14, 15]. In our previous study, we reported that CAFs could have a more significant effect on the motility of GC cells than paired normal fibroblasts (NFs) [13]. However, the exact molecular mechanisms have not been elucidated. Several researchers have reported that the distinct gene expression patterns of CAFs differ from those of NFs in various solid tumors [16–20]. They reported that CAF-specific molecules vary according to the cancer type; however, the exact mechanism of the interaction between CAFs and cancer cells has not been elucidated in DGC.

In 2003, tubulointerstitial nephritis antigen-like 1 (TINAGL1), also known as adrenocortical zonation factor-1 (AZ-1) and lipocalin-7 (LCN7), was newly observed in adrenocortical cells [21]. Li et al. reported that TINAGL1 is a new member of matricellular proteins and can activate integrins, including α1β1, α2β1, and α5β1 [22]. Additionally, TINAGL1 enhances the activation of integrins induced by extracellular matrix (ECM) proteins, including fibronectin and collagen. Recently, the correlation between TINAGL1 and several cancers was reported [23–26]. TINAGL1 was suggested as a potential therapeutic target to inhibit metastasis in non-small cell lung cancer [23]. During hepatocellular carcinogenesis, TINAGL1 increases VEGF expression via the TGF-β/Smad3 signaling pathway [24]. In gastric cancer, upregulated TINAGL1 enhances the expression of matrix metalloproteinases (MMPs), especially MMP2, via the JNK signaling pathway and promotes tumor progression and metastasis [26]. However, *Tinagl1* suppresses tumor progression and metastasis by inhibiting integrin/FAK and EGFR downstream signaling pathways in triple-negative breast cancer [25]. The functional role of TINAGL1 in cancer is still controversial. In addition, previous studies have focused on the role of cancer cell-derived TINAGL1, but the origin of intratumor TINAGL1 remains uncertain. It has further been shown that fibroblast-derived TINAGL1 could play an essential role in wound healing [27, 28]; thus, CAFs could be a source of TINAGL1 in the stroma of fibrotic tumors.

In this study, we identified TINAGL1 as a CAF-specific molecule in DGC using multi-omics analysis. We evaluated the role of CAF-secreted TINAGL1 in tumor progression and prognosis. Based on our results, we suggest stromal TINAGL1 as an independent prognostic marker and novel therapeutic target for DGC patients.

## Materials and methods

### Cell culture

We purchased the gastric cancer cell lines MKN45, KATO-III, SNU601, and SNU668 from the Korean Cell Line Bank (Seoul, Korea). The cells were cultured in RPMI-1640 (Cytiva) supplemented with 10% FBS (Hyclone) and 1% penicillin–streptomycin (Gibco; Thermo Fisher Scientific). Cells were incubated at 37 °C in a humidified atmosphere containing 5% CO_2_.

### Isolation of primary fibroblasts

Human gastric cancer specimens were obtained from patients undergoing tumor resection at Ajou University Hospital (Suwon, Korea). CAFs and paired NFs were isolated from fresh GC patient specimens, as described previously [13]. Cells were cultured in DMEM/high glucose (DMEM; Cytiva) supplemented with 10% FBS and 1% penicillin–streptomycin. Primary cultured fibroblasts were used within seven passages considering the difficulty of maintaining their features for a long period of time after isolation from tissues.

### Preparation of conditioned media

NFs or CAFs at 80% confluency were incubated in serum-free DMEM for 48 h. The cell culture medium was collected in a 15-ml conical tube and centrifuged at 2000 rpm at 4 °C for 10 min. The supernatant was transferred to a new 15-ml conical tube and stored at − 20 °C until further use.

### Co-culture and treatment

SNU601 cells were seeded in a 6-well plate at 5.5 × 10^5^ cells/well. MKN45 and KATO-III cells were seeded in 6-well plates at a density of 3 × 10^5^ cells/well. NFs or CAFs were seeded in a 0.4-μm pore-sized transwell chamber (SPL). Cancer cells were incubated in serum-free DMEM overnight. The chambers were then inserted into cancer cell-seeded plates. The cells were cultured in DMEM supplemented with 5% FBS. Cancer cells, cultured in 5% FBS-DMEM, were treated with recombinant human TINAGL1 (R&D Systems), fibronectin (BD Biosciences), and PF-573228 (AdooQ Bioscience).

### Transwell migration assay

SNU601, MKN45, and KATO-III cells were seeded in an 8.0-μm pore-sized transwell chamber (SPL) at 2 × 10^4^ cells/well. NFs or CAFs were seeded in 24-well plates at 2 × 10^4^ cells/well. Cancer cells were incubated in serum-free DMEM overnight. The chambers were then inserted into a fibroblast-seeded plate. DMEM supplemented with 10% FBS was added to the bottom chamber as a chemoattractant. After incubation for 48 h, cells in the transwell chamber were fixed with methanol and stained with hematoxylin and eosin (H&E). The non-migrated cells were wiped off with a cotton swab, and the migrated cells were visualized under a phase-contrast microscope and manually counted in three randomly selected fields (magnification, × 100).

### Soft agar assay

A 6-well plate was coated with 1 ml of 1.0% agarose gel (Sigma-Aldrich). 5 × 10^3^ SNU601 cells with or without 2.5 × 10^3^ fibroblasts were seeded in 0.5% agarose gel. The medium was changed every 3 days. After 25 days, the colonies in the gel were stained with 0.01% crystal violet solution, and the number and area of colonies were counted using the ImageJ software. The 6-well plate was coated with 1 ml of 1.0% agarose gel. 5 × 10^3^ cells of SNU601 were seeded on 0.5% agarose gel. The medium, with or without recombinant TINAGL1 protein (100 ng/ml), was changed every 3 days. After 20 days, the colonies in the gel were stained with 0.01% crystal violet solution, and the size of the five largest colonies in each well was measured using the ImageJ software.

### Cell proliferation assay

SNU601 cells were seeded in a 96-well plate at a density of 5 × 10^3^ cells/well and cultured with DMEM, NF-, or CAF-CM supplemented with 5% FBS for 0, 24, 48, and 72 h. After incubation, 10 μl of Qunati-Max™ (Biomax) was added to each well, and the cells were incubated for 1 h and 30 min at 37 °C. The absorbance at 450 nm was measured using a microplate reader. All experiments were performed in triplicate.

### Transcriptome analysis

Total RNA from three pairs of DGC primary fibroblasts was extracted using an RNeasy Mini Kit (QIAGEN), following the manufacturer’s instructions. A detailed clinical information for the patients is indicated in Additional file 2: Table S1. Total RNA sequencing was performed using the Illumina NextSeq 500 platform (Illumina). Gene set enrichment analysis was performed for differentially expressed genes (DEGs).

### Proteomic analysis

Two CAF and two paired NF-CM were prepared as described above. The samples were concentrated and digested using trypsin. LC–MS/MS was also performed. Protein intensities were normalized using Normalyzer [29], and log_2_ fold-changes were calculated.

### RNA isolation and reverse transcription PCR (RT-PCR)

Total RNA was extracted using an RNeasy Mini Kit (QIAGEN), following the manufacturer’s instructions. The concentration of RNA was measured using Nanodrop (Bio-Rad), and 1 μg of RNA was used for cDNA synthesis. cDNA synthesis was performed using an iScript™ cDNA Synthesis Kit (Bio-Rad) according to the manufacturer’s instructions. RT-PCR was performed using AccuPower® Taq PCR PreMix (Bioneer), following the manufacturer’s instructions. The cDNA mixture was then subjected to 35 cycles of PCR amplification. The primer sequences used for RT-PCR are listed in Additional file 3: Table S2. The PCR products were mixed with Dyne Loading STAR (Dyne Bio) and visualized using agarose gel electrophoresis.

### Quantitative RT-PCR (qRT-PCR)

qRT-PCR was performed using iQ™ SYBR® Green Supermix (Bio-Rad), according to the manufacturer’s instructions, and the assays were carried out using the CFX Real-Time PCR Detection System (Bio-Rad). The primer sequences used for qRT-PCR are listed in Additional file 4: Table S3. All experiments were performed in duplicate. The intensity of the fluorescent dye was determined and the expression levels of each mRNA were normalized to that of *GAPDH.*

### Enzyme-linked immunosorbent assay (ELISA)

The TINAGL1 concentration in the CM of fibroblasts was measured using the Human TINAGL1/Lipocalin 7 ELISA Kit (LSBio) following the manufacturer’s instructions. NF- and CAF-CM were prepared as previously above. All experiments were performed in duplicate.

### Western blotting

Cells were washed with PBS and lysed with SDS lysis buffer. Frozen tissues were lysed using T-PER Tissue Protein Extraction Reagent (Thermo Fisher Scientific). Protein concentrations were determined using the Bradford assay (Bio-Rad). Equal amounts of protein from each sample were resolved by SDS-PAGE and transferred onto PVDF membranes (Millipore). Immunoblots were blocked by incubation in 5% skim milk in TBS-T (0.1% Tween 20) for 1 h at 25 °C. Membranes were incubated with the following primary antibodies: anti-TINAGL1 (1:1000; 12077-1-AP, Proteintech), anti-pFAK (1:1000; #3283; Cell Signaling Technology), anti-FAK (1:1000; #3285; Cell Signaling Technology), β-actin (1:10,000; sc-47778, Santa Cruz Biotechnology), anti-Twist (1:1000; ab49254, Abcam), anti-E-cadherin (1:500; #13-1700; Invitrogen), anti-N-cadherin (1:1000, ab18203, Abcam), anti-integrin β1 (1:1000; ab30394, Abcam), anti-integrin αv (1:1000; #4711, Cell Signaling Technology), anti-integrin α5 (1:1000; #4705, Cell Signaling Technology), and anti-GAPDH antibodies (1:20,000; Abc-1001, AbClon), followed by their corresponding HRP-conjugated secondary antibodies, anti-mouse (1:5000: #115-035-003, Jackson ImmunoResearch Labs) and anti-rabbit antibodies (1:4000; #Abc-5003, AbClon). Proteins were detected using AbSignal (Abclone).

### Immunoprecipitation

Immunoprecipitation (IP) for SNU601 cells was performed using the Pierce™ Co-Immunoprecipitation Kit (Pierce; Thermo Fisher Scientific) following the manufacturer’s instructions. Briefly, the cells were lysed in IP Lysis/Wash buffer. The cell lysate was incubated with 5 µl of anti-TINAGL1 antibody (12077-1-AP, Proteintech) for 2 h at 4 °C. Then, the protein complexes were collected using elution buffer and analyzed by immunoblotting.

### Immunohistochemistry (ICC)

MKN45 (20 × 10^4^), KATO-III (10 × 10^4^), SNU601 (10 × 10^4^), and SNU668 (6 × 10^4^) cells were seeded on 12-mm cover glasses. The next day, the cells were fixed with 4% paraformaldehyde for 10 min at 25 °C and blocked with 5% BSA in PBS for 30 min at 25 °C. The cells were incubated with an anti-integrin β1 antibody (1:200; ab30394, Abcam) overnight at 4 °C. The slides were then incubated with anti-mouse-Alexa488 antibody (1:1000; 115-545-146, Jackson ImmunoResearch Labs) and Hoechst 33258 (Sigma-Aldrich) at 25 °C for 30 min. The cells were then mounted on glass slides, and all images for ICC were acquired at the same exposure times.

### Phospho explorer antibody array

For the Phospho Explorer antibody array, SNU601 cells were seeded in a 6-well plate and co-cultured with NFs or CAFs. The antibody array was performed using the Phospho Antibody array kit (Fullmoon Biosystems), as previously described [30, 31].

### Small interfering RNA (siRNA)

ON-TARGETplus non-targeting control pool (D-001810-10-05; Dharmacon) and human TINAGL1 siRNA (L-008373-00-0005, Dharmacon), a pool of siRNA containing four sequences with patented modifications to reduce off-target effects, were used. siRNA was transfected into CAF47 cells at a concentration of 30 nM using Lipofectamine RNAiMAX (Invitrogen). After 3 days, the medium was replaced, and the cells were used for further study. TINAGL1 levels were validated by RT-PCR and western blotting.

### Cell immortalization and short hairpin RNA (shRNA)

The pBABE-neo-hTERT plasmid (#1774, Addgene) was used to generate immortalized fibroblasts. NF47 and CAF47 cells were infected with retroviruses and passaged. hTERT levels were validated using RT-PCR. The pGFP-C-shLenti plasmid containing anti-*TINAGL1* (TL307228, Origene) or scrambled negative control sequences (TR30023, OriGene) were used to generate stable cell lines. Immortalized CAFs were infected with lentivirus, and GFP-expressing cells were selected. TINAGL1 levels were validated by RT-PCR.

### Dual RNA in situ hybridization (RNA-ISH)

RNA-ISH was performed using the RNAscope™ 2.5 HD Duplex Reagent Kit (ACD) following the manufacturer’s instructions. Human FFPE tissues were cut into 5-µm sections, and target retrieval was performed. The slides were then incubated with the target probe for 2 h at 40 °C. The slides were then subjected to signal amplification followed by signal detection. The probes used for RNA-ISH were Hs-COL1A1 (401891; ACD) and Hs-TINAGL1-C2 (857221-C2; ACD). All stained slides were scanned using a slide scanner (Axioscan Z1; Carl Zeiss Microscopy GmbH) at the three-dimensional immune system image core facility. Three regions of interest (ROI) were imaged and randomly acquired for each tissue (magnification, × 400), and the number of dots was counted manually.

### Immunohistochemistry (IHC)

FFPE tissues were cut into 4-µm-thick sections, and antigen retrieval was performed using Tris–EDTA (pH 9.0). The slides were blocked in 20% Aqua Block Buffer solution (Abcam) and incubated with the primary antibody overnight at 4 °C. The slides were then incubated with the secondary antibody at 25 °C for 30 min, followed by detection using a DAB Substrate Kit (Abcam). The antibodies used for immunohistochemistry were anti-cytokeratin (1:100; NB600-557, Novus Biologicals), anti-E-cadherin (1:1000; #3195, Cell Signaling Technology), and anti-rabbit antibodies (1:100, #Abc-5003, AbClon). All antibodies were diluted in an antibody diluent for IHC (BD Biosciences).

### Public data

We analyzed the GSE15459 and The Cancer Genome Atlas Stomach Adenocarcinoma (TCGA-STAD) datasets using the R 4.1.1 software program. The GSE15459 dataset includes mRNA expression data from 200 primary gastric tumors, and the TCGA-STAD dataset includes mRNA expression data from 443 primary gastric tumors. The hazard ratio and *P* value were calculated using the multivariate Cox regression model and are indicated on a Kaplan–Meier plot.

### Animal model study

Animal care and handling were carried out in accordance with the guidelines of the Ajou University School of Medicine Institutional Animal Care and Use Committee Committee (IACUC). All animal experiments were approved by the Animal Research Committee of the institution (2015-0069). Five-week-old male BALB/c nude mice (DBL, Eumseong-gun, Korea) were used for the animal model study.

To generate intraperitoneal xenograft tumors, mice were subcutaneously injected with 1 × 10^6^ SNU601 cells with or without 1 × 10^6^ fibroblasts suspended in 100 μl of PBS. To generate subcutaneous xenograft tumors, mice were subcutaneously injected with 2 × 10^6^ SNU601 with or without 1 × 10^6^ cells of fibroblasts suspended in 100 μl of PBS with 50% Matrigel Basement Membrane Matrix Growth Factor Reduced (Corning). Tumor volume was measured three times per week and calculated using the formula (length × width2)/2. Tumor tissues were collected, weighed, fixed using 10% neutral buffered formalin (NBF), and embedded in paraffin for further analysis.

### Statistical analysis

Statistical analyses were performed using the R 4.1.1 software program and GraphPad Prism 9. Data are presented as the mean ± standard error (SE). The means of the two groups were compared using the Wilcoxon rank-sum exact test or the paired t-test. The Kruskal–Wallis test, followed by Dunn’s post-hoc test, was used to compare means across three groups. The Cox regression model was used to examine correlations with overall survival. Statistical significance was set at *P* < 0.05.

## Results

### Integrated transcriptome and proteomics analysis reveals TINAGL1 as a specific molecule produced by CAFs

**Fig. 1:**
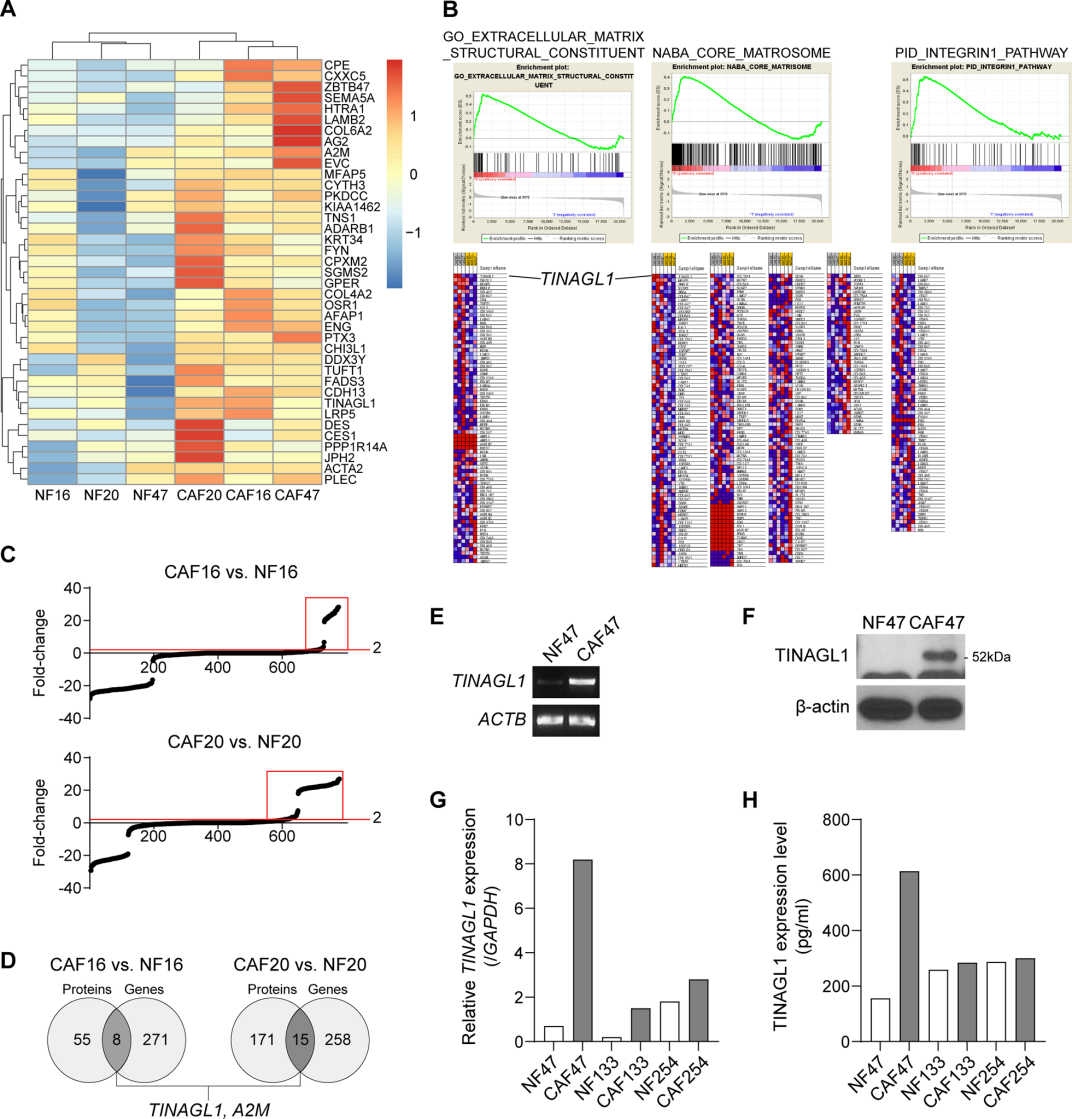
TINAGL1 is a CAF-specific secretory molecule in DGC. **A** The heatmap represents the top-40 CAF-up-regulated genes, which were commonly increased in three NF-CAF pairs (fold-change > 2). **B** Gene set enrichment analysis for CAF-up-regulated genes. **C** Proteomic analysis for paired NF- and CAF-conditioned media. The red box indicates the enriched proteins in CAFs exhibiting over twofold-change. **D** The Venn diagram shows CAF-specific molecules up-regulated at the gene and protein levels simultaneously. **E–H** TINAGL1 gene and protein levels were validated in NF-CAF pairs by RT-PCR (**E**), western blotting (**F**), qRT-PCR (**G**), and ELISA (**H**)

**Fig. 2:**
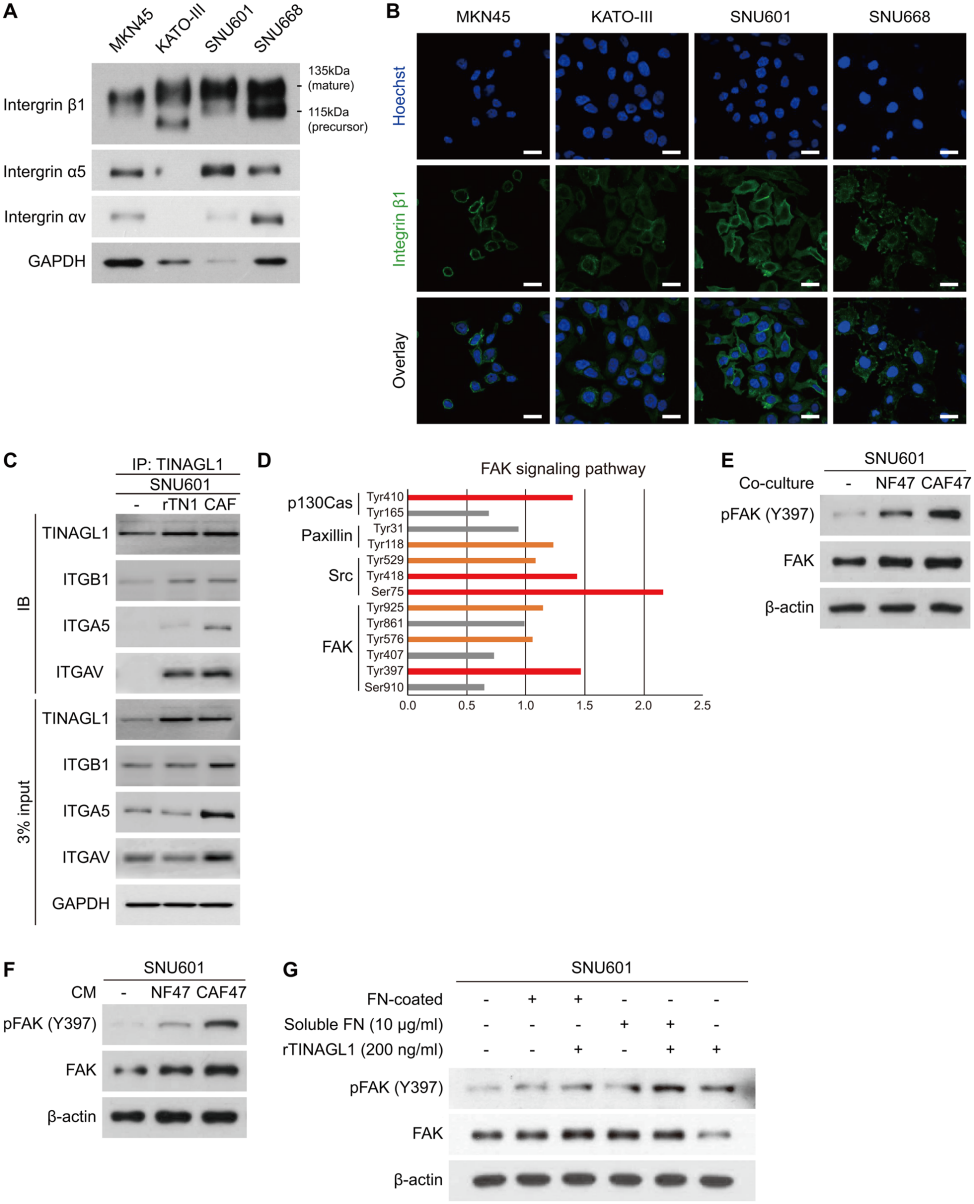
TINAGL1 interacts with integrins and activates the FAK signaling pathway. **A** Western blotting for integrin subunit levels in MKN45, KATO-III, SNU601, and SNU668 cells. **B** Representative images of immunocytochemistry staining for integrin β1 (green) in DGC cell lines (blue, nucleus; scale bars, 20 μm). **C** Immunoprecipitation assay was performed in SNU601 cells treated with recombinant TINAGL1 (rTINAGL1) or co-cultured with CAF47 (CAF). **D** Phospho Explorer antibody array for NF47- or CAF47-co-cultured SNU601 cells (orange, fold-change > 1; red, fold-change > 1.3). **E**, **F** Western blotting for FAK phosphorylation in fibroblasts co-cultured (**E**) or CM-treated (**F**) SNU601 cells. **G** Western blotting for FAK phosphorylation in fibronectin (FN) or TINAGL1-treated SNU601 cells

We previously reported that CAFs increased the aggressiveness of DGC cells to a greater extent than NFs [13]. To identify a CAF-specific molecule that contributes to their tumor-promoting effect, we integrated transcriptome and proteomic analysis data (Additional file 1: Fig. S1). First, we performed transcriptome analysis with three NF-CAF pairs derived from DGC patients’ tissues (Additional file 2: Table S1). RNA-seq revealed several commonly increased CAF-specific genes among each pair (Fig. 2A). Gene set enrichment analysis (GSEA) for differentially expressed genes (DEGs; |fold-change|> 2) revealed that gene sets, including the integrin1 pathway, core matrisome, and extracellular matrix structural constituents, were increased in CAFs (Fig. 1B). Next, we performed proteomic analysis for NF- or CAF-conditioned media (CM) using liquid chromatography-tandem mass spectrometry (LC–MS/MS) and found that paired NFs and CAFs derived from DGC patient tissues exhibited varying expression patterns of secreted proteins (Fig. 1C). Finally, we identified CAF-upregulated extracellular molecules at both the protein and gene levels in two pairs, TINAGL1 and A2M (fold-change > 2; Fig. 1D, Additional file 5: Table S4, Additional file 6: Table S5). Furthermore, *TINAGL1* was highly ranked among CAF-upregulated gene sets (Fig. 1B). Thus, we focused on the role of TINAGL1 in DGC development. TINAGL1, also known as AZ-1 or LCN7, is a secretory protein that has been reported to be a ligand for integrins [22]. Increased TINAGL1 protein and gene expression in CAFs were validated with the NF47-CAF47 pair, which was excluded from the proteomic analysis (Fig. 1E–H). Furthermore, CAF47 showed the highest expression of both the *TINAGL1* gene and TINAGL1 protein (Fig. 1G, H). These results indicate that NFs and CAFs exhibit distinct molecular features, and TINAGL1 is a CAF-specific secretory protein in DGC.

### CAF-secreted TINAGL1 can interact with integrin and activate the FAK signaling pathway in DGC cells

We found that TINAGL1 was a CAF-specific secretory protein. Thus, we examined the functional role of TINAGL1 in DGC. We hypothesized that there is a receptor on DGC cells that allows cancer cells to respond to stimulation from CAFs. It was reported that TINAGL1 protein binds to integrins αvβ1 and α5β1 [25]. We thus confirmed these integrin subunit levels in several DGC cell lines. Among them, SNU601 cells exhibited the highest level of integrins (Fig. 2A, Additional file 1: Fig. S3A). Furthermore, the membrane expression of integrin β1, a common subunit of those two combinations, was also the highest in SNU601 cells (Fig. 2B). We thus selected SNU601 cells to investigate the functional significance of the TINAGL1-integrin interaction and its downstream signaling pathway. We confirmed interaction between TINAGL1 and these integrin subunits using immunoprecipitation (Fig. 2C). Our data also revealed that the protein levels of TINAGL1 increased in both recombinant TINAGL1-treated and CAF-co-cultured cancer cells. These results suggest that secreted TINAGL1 from CAFs could bind to the plasma membrane of cancer cells. Moreover, recombinant or CAF-derived TINAGL1 protein interacts with integrin subunits, especially β1, in cancer cells. We assume that integrin β1 is primarily involved in the TINAGL1-mediated signaling pathway.

Next, we carried out a Phospho-Explorer antibody array with NF- or CAF-co-cultured SNU601 cells to identify the downstream signaling pathways activated by CAF-derived TINAGL1. We confirmed that phosphorylation of proteins associated with the FAK signaling pathway was increased in SNU601 cells co-cultured with CAFs compared to cells co-cultured with NFs (Fig. 2D). Phosphorylation of FAK signaling pathway proteins is a well-known consequence of integrin activation [32]. The promotion of FAK phosphorylation was validated using western blotting (Fig. 2E, F, Additional file 1: Fig. S3B, C). FAK in SNU601 cells was activated by both CAF-CM treatment and co-culture. Additionally, FAK phosphorylation in other DGC cells with low integrin expression, namely, MKN45 and KATO-III cells, did not occur (Additional file 1: Fig. S2A). Li et al. reported that TINAGL1, along with other ECM proteins, induces the accumulation of ligand-activated integrins on plasma membranes and increases intracellular signaling [22]. However, Shen et al. reported that TINAGL1 competes with fibronectin to interact with the integrin β1 subunit and inhibits FAK phosphorylation in triple-negative breast cancer cells [25]. To determine whether TINAGL1 promotes FAK phosphorylation, we treated SNU601 cells with recombinant human TINAGL1 protein alone or with fibronectin (Fig. 2G, Additional file 1: Fig. S3D). In SNU601 cells, both plate-coated and soluble fibronectin induced FAK phosphorylation. Moreover, TINAGL1 treatment enhanced FAK activation by fibronectin and induced FAK phosphorylation alone. Taken together, CAF-derived TINAGL1 interacts with integrin and activates the FAK signaling pathway in DGC cells.

### TINAGL1-high CAFs can more contribute to the aggressiveness of DGC cells than NFs

**Fig. 3:**
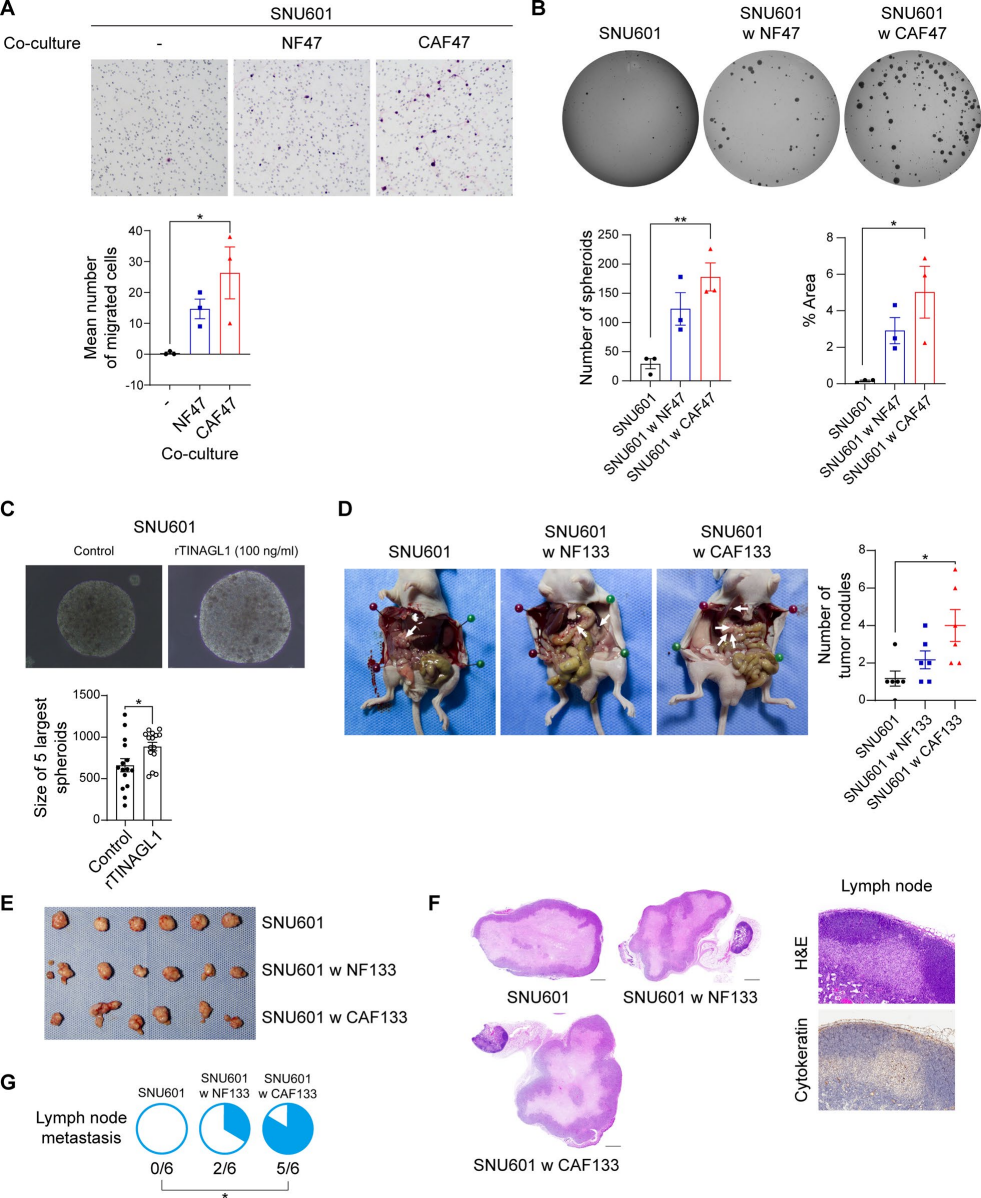
CAF-secreted TINAGL1 increased the aggressiveness of DGC cells*.* **A** Representative images and graphs of the transwell migration assay of SNU601 cells with or without fibroblasts (magnification, × 100). Data were analyzed using the Kruskal–Wallis test with Dunn’s test. **B** Representative images and graph of the soft agar assay of SNU601 cells with or without fibroblasts. The number of spheroids and percentage area were counted using the ImageJ software. Data were analyzed using ANOVA with Bonferroni correction. **C** Representative images and graphs of the soft agar assay of SNU601 cells with or without recombinant TINAGL1 treatment (rTINAGL1). Data analyzed using the Wilcoxon test. **D** Representative images and graphs of intraperitoneally injected SNU601 xenograft tumors (n = 6 per group; arrow, tumor nodules). Data were analyzed using the Kruskal–Wallis test with Dunn’s test. **E**, **F** Representative images of subcutaneously injected SNU601 xenograft tumors (**E**) and H&E staining of the entire tumor and cytokeratin staining of harvested inguinal lymph nodes (**F**). Scale bars, 1000 μm. **G** Pie chart shows the number of tumors with lymph node metastasis. Data were analyzed using Fisher’s exact test. *P < 0.05, **P < 0.01

Next, we examined whether CAF-derived TINAGL1 contributed to the aggressiveness of DGC cells in vitro and in vivo. First, we performed a transwell migration assay. Although co-culture with NFs or CAFs increased the migration ability of SNU601 cells, only CAFs significantly increased cancer cell migration (Fig. 3A). TINAGL1-high CAFs did not affect MKN45 and KATO-III cells, as seen with FAK phosphorylation (Additional file 1: Fig. S2B, C). In the soft agar assay, only CAFs enhanced the tumorigenesis of SNU601 cells (Fig. 3B). Treatment with recombinant TINAGL1 also enhanced the tumorigenic ability of SNU601 cells (Fig. 3C). There were no differences in cancer cell proliferation induced by NF- and CAF-CM (Additional file 1: Fig. S4A). These results indicate that TINAGL1-high CAFs enhanced migration and tumorigenesis of DGC cells but not their proliferation in vitro.

To understand the role of CAF-derived TINAGL1 better, SNU601 cells were injected into BALB/c nude mice with or without NFs or CAFs. In the intraperitoneal xenograft model, co-injection with CAFs significantly increased the number of peritoneal tumor nodules (Fig. 3D, Additional file 1: Fig. S4B). Interestingly, subcutaneous xenograft tumors with CAFs exhibited morphologically invasive tumor surfaces, and some of them exhibited lymph node metastasis (Fig. 3E–G). However, co-injection with CAFs did not enhance tumor growth (Additional file 1: Fig. S4C, D). We used cytokeratin as a cancer cell marker and E-cadherin as an epithelial marker in epithelial-to-mesenchymal transition (EMT). In several tumors, cytokeratin-positive cancer cells were detected adjacent to inguinal lymph nodes, especially in CAF-co-injected tumors (Fig. 3F). Moreover, cytokeratin-positive cancer cells at the tumor margin site exhibited reduced E-cadherin expression in CAF-co-injected tumors (Additional file 1: Fig. S4E). These results indicate that TINAGL1-high CAFs increased tumorigenesis, invasion, and metastasis of DGC but not tumor growth in vivo.

### Inhibition of the TINAGL1/integrin/FAK axis can alleviate CAF-induced aggressiveness of DGC cells

**Fig. 4:**
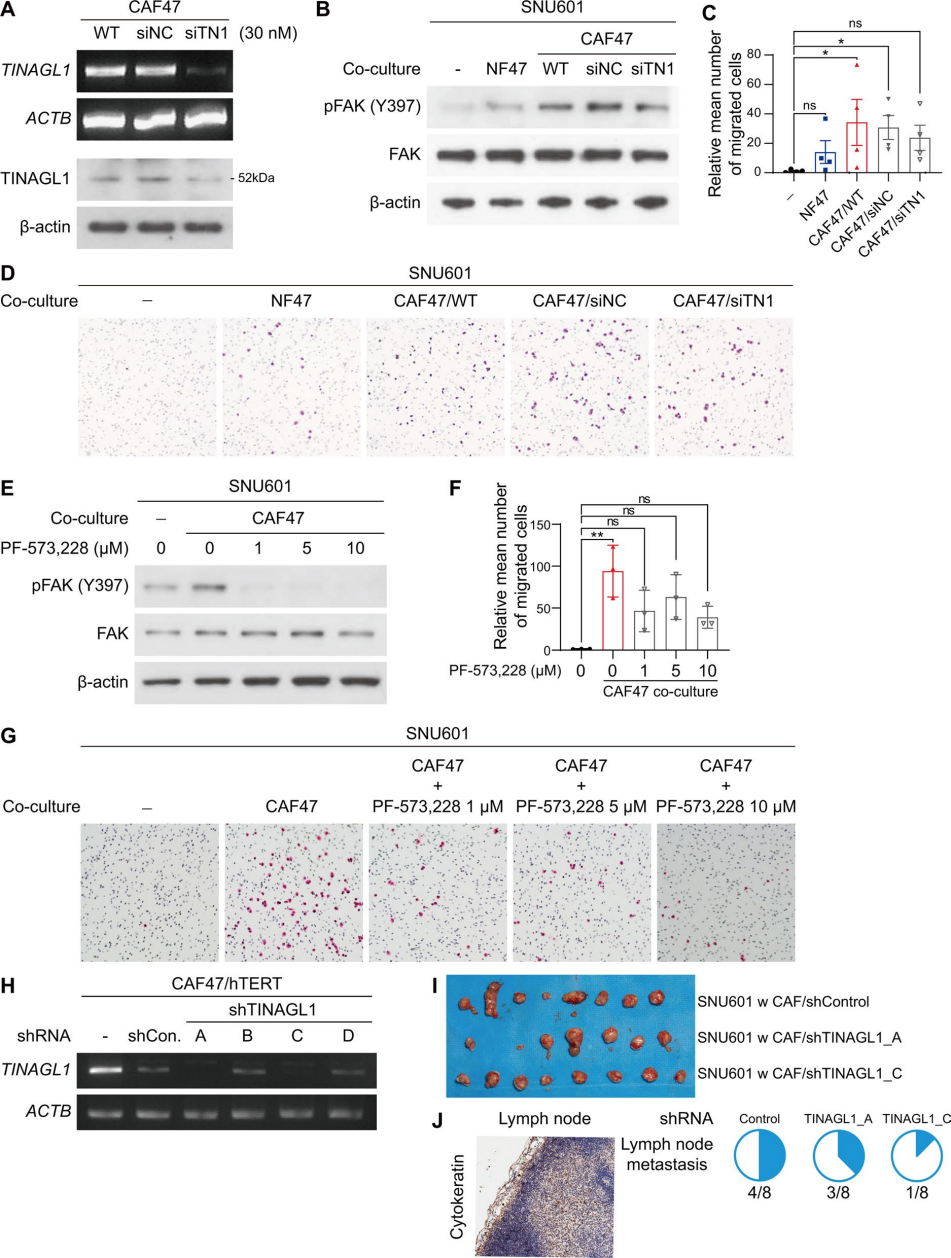
Inhibition of the TINAGL1/integrin/FAK axis decreased the CAF-induced aggressiveness of DGC. **A** TINAGL1 gene and protein levels in siRNA-transfected CAF47 by RT-PCR and western blotting. **B** Western blotting for FAK phosphorylation in siRNA-transfected CAF47-co-cultured SNU601 cells. **C**, **D** Graph (**C**) and representative images (**D**) of the transwell migration assay of siRNA-transfected CAF47-co-cultured SNU601 cells (magnification, × 100). Data were analyzed using the Kruskal–Wallis test with Dunn’s test. **E** Western blotting for FAK phosphorylation in PF-573,228-treated SNU601 cells. **F**, **G** Graph (**F**) and representative images (**G**) of the transwell migration assay of PF-573,228-treated SNU601 cells (magnification, × 100). Data were analyzed using the Kruskal–Wallis test with Dunn’s test. **H** RT-PCR for *TINAGL1* expression in shRNA-transfected immortalized CAF47. **I** Representative image for subcutaneously injected SNU601 xenograft tumors. The small tissue clusters under each large tumor tissue are metastasized inguinal lymph nodes. **J** Representative image for cytokeratin staining, and the pie chart shows the number of tumors with lymph node metastasis. *P < 0.05, **P < 0.01

To investigate the effect of CAF-derived TINAGL1, we used TINAGL1-targeting siRNA, which reduced TINAGL1 protein and mRNA levels effectively (Fig. 4A). In addition, TINAGL1-deficient CAFs did not induce FAK phosphorylation and migration in cancer cells to the same extent as wild-type or negative control siRNA-treated CAFs (Fig. 4B–D, Additional file 1: Fig. S3E). TINAGL1 siRNA also slightly reduced the expression of the mesenchymal marker Twist in cancer cells (Additional file 1: Fig. S6A). Next, we inhibited FAK phosphorylation using the small-molecule inhibitor PF-573228, which disrupted CAF-induced FAK signaling activation and migration in cancer cells (Fig. 4E–G, Additional file 1: Figs. S3F and S6B).

To assess the long-term inhibitory effect of TINAGL1, TINAGL1-deficient stable CAF cell lines were used. Due to the limited growth potential of primary cultured cells, we established immortalized patient-derived fibroblast cell lines by overexpressing *hTERT*. The immortalized fibroblast cell lines exhibited similar levels of TINAGL1 and similar tumor-promoting effects as wild-type cells (Additional file 1: Fig. S5A, B). We developed stable CAF/shTINAGL1 cell lines using immortalized CAFs. Although CAF/shControl cells exhibited reduced *TINAGL1* expression compared to immortalized CAFs, CAF/shTINAGL1_A and C exhibited completely inhibited *TINAGL1* expression (Fig. 4H). We generated a subcutaneous xenograft tumor model using TINAGL1-deficient CAFs. Inhibition of TINAGL1 in CAFs slightly reduced tumor growth and volume (Fig. 4I, Additional file 1: Fig. S6C, D). In addition, lymph node metastasis decreased, and E-cadherin expression at the tumor margin site increased, especially in CAF/shTINAGL1_C co-injected tumors (Fig. 4J, Additional file 1: Fig. S6E).

Taken together, CAF-derived TINAGL1 may contribute to FAK signaling and metastasis in DGC. Inhibition of the TINAGL1/integrin/FAK axis can alleviate CAF-induced tumor progression in DGC.

### Stromal TINAGL1 expression is significantly correlated with the oncologic outcome of human DGC

**Fig. 5:**
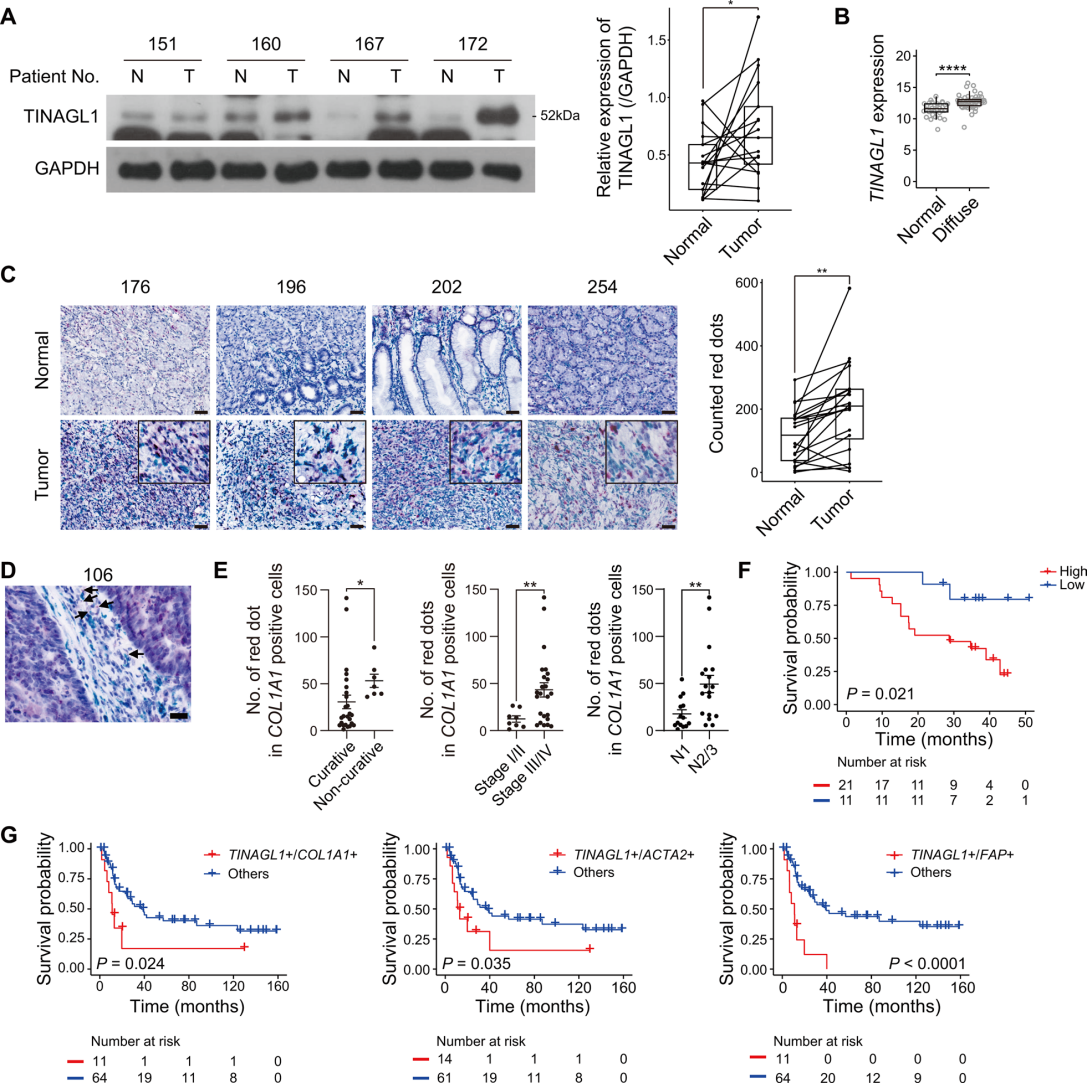
Stromal *TINAGL1* expression is significantly correlated with the oncologic outcomes in DGC patients. **A** Representative western blotting image of TINAGL1 expression in paired normal and tumor tissues (n = 17). The graph indicates the relative TINAGL1 expression to GAPDH. Data were analyzed using a paired t-test. **B** *TINAGL1* expression in normal and diffuse-type tumor tissues from the TCGA-STAD dataset. Data were analyzed using the Wilcoxon test. **C** Representative images and graph of RNA-ISH in paired normal tumor tissues (n = 20; red, *TINAGL1*; green, *COL1A1*; scale bars, 50 μm). Data were analyzed using a paired t-test. **D** Arrows indicate red dots in *COL1A1*-positive cells (scale bar, 20 μm). **E** Graph indicates stromal *TINAGL1* expression of DGC patient tumor tissues using RNA-ISH and expression according to resection, stage, and lymph node metastasis status (n = 32). Data were analyzed using the Wilcoxon test. **F** Kaplan–Meier plot for stromal *TINAGL1* expression in DGC patients (n = 32). **G** Kaplan–Meier plots for *TINAGL1* and *COL1A1*, *ACTA2*, or *FAP* expression in DGC patients from the GSE15459 dataset (n = 75). *P < 0.05, **P < 0.01, ****P < 0.0001

Finally, we estimated whether stromal TNAGL1 expression correlated with poor prognosis in DGC. We investigated TINAGL1 protein and gene expression in bulk tumors. Western blotting of paired normal and tumor tissues showed that the TINAGL1 protein levels were increased in tumor tissues (Fig. 5A). *TINAGL1* expression in the TCGA-STAD dataset was also increased in DGC tumor tissues (Fig. 5B). To investigate stromal *TINAGL1* expression, we performed dual RNA in-situ hybridization (RNA-ISH) in FFPE tissues and counted the number of red dots (*TINAGL1*) detected in *COL1A1*-positive cells (Fig. 5C–F). The colocalization of *TINAGL1* and *COL1A1*, a fibroblast marker, was observed and more pronounced in the tumor tissues (Fig. 5C). We then estimated the correlation between stromal *TINAGL1* expression and prognosis in 32 DGC patients. Stromal *TINAGL1* expression was significantly increased in non-curative resection, stage III/IV, and N3 DGC patients (Fig. 5D, E). We also investigated the correlation between the expression of *TINAGL1* and integrin subunits based on bulk RNA-sequencing data. Among those subunits, *ITGB1* had the strongest correlation with *TINAGL1* expression in both datasets (Additional file 1: Fig. S7A, B).

Patients with high stromal *TINAGL1* expression on RNA-ISH exhibited a poor overall survival rate (Fig. 5F). We further investigated the correlation between stromal *TINAGL1* expression and overall survival using several public datasets. In the GSE15459 dataset, co-expression of *TINAGL1* and several CAF marker genes, including *COL1A1,* actin alpha 2, smooth muscle (*ACTA2*), and fibroblast activation protein alpha (*FAP*), was correlated with poor prognosis in DGC patients but not in intestinal-type patients (Fig. 5G, Additional file 1: Fig. S8A). In addition, co-expression of *TINAGL1* and several CAF marker genes are independent prognostic indicators for the overall survival of patients with DGC (Table 1, Additional file 7: Table S6, Additional file 8: Table S7). In the TCGA-STAD dataset, co-expression of *TINAGL1* and several CAF marker genes were not statistically correlated with overall survival in both the diffuse and intestinal type, but indicated worse prognosis in the diffuse type (Additional file 1: Fig. S8B).

**Table 1:** *TINAGL1* and *COL1A1* expression is associated with overall survival of DGC patients in the GSE15459 dataset

Variable	n (%)	Univariate		Multivariate	
		HR (95% CI)	*P*-value	HR (95% CI)	*P*-value
*TINAGL1/COL1A1*					
Others	64 (85.3)	–		–	
TINAGL1 + /COL1A1 +	11 (14.7)	2.41 (1.09–5.28)	0.029	3.78 (1.37–10.41)	0.010
Age					
> 65	31 (41.3)	–		–	
65 ≥	44 (58.7)	0.80 (0.43–1.51)	0.498	0.77 (0.36–1.68)	0.516
Gender					
Female	39 (52.0)	–		–	
Male	36 (48.0)	1.55 (0.83–2.90)	0.170	0.93 (0.45–1.94)	0.845
Stage					
I	9 (12.0)	–		–	
II	12 (16.0)	7.98 (0.98–65.10)	0.052	10.87 (1.24–95.02)	0.031
III	31 (41.3)	9.49 (1.25–72.24)	0.030	12.82 (1.57–104.51)	0.017
IV	23 (30.7)	33.96 (4.36–264.71)	0.001	57.30 (6.64–494.59)	< 0.001
Subtype					
Metabolic	16 (26.2)	–		–	
Proliferative	12 (19.7)	1.51 (0.54–4.20)	0.427	0.72 (0.25–2.08)	0.541
Mesenchymal	33 (54.1)	1.60 (0.71–3.63)	0.256	0.98 (0.40–2.41)	0.968

Cox regression model

*HR* hazard ratio, *CI* confidence interval

Taken together, stromal TINAGL1 expression was increased in tumor tissues of patients with DGC, and it can be an independent prognostic indicator for overall survival.

## Discussion

Despite clinical evidence about the clinical role of CAFs in DGC [11, 13], the underlying mechanisms related to CAF-induced progression of DGC are still unclear. In the present study, we found that a specific molecule originating from CAFs, TINAGL1, influences the progression of DGC through activation of the Integrinβ1/FAK signaling pathway. Moreover, TINAGL1 was more expressed in cancer tissues of human DGC than in paired normal gastric tissues. The expression of TINAGL1 in the fibroblasts of DGC was significantly correlated with the progression of the disease and poor prognosis for patients.

In this study, we suggest CAF-specific makers based on transcriptome and proteomic analysis for paired fibroblasts isolated from normal and cancer tissues in patients with DGC. Although the origin of CAFs is still not fully understood, quiescent fibroblasts in normal tissues are considered a major source of CAFs [15]. Therefore, exploration of the functional or molecular differences between CAFs isolated from tumors and fibroblasts from paired normal tissues is critical to demonstrate CAF-specific mechanisms for tumor progression. Based on transcriptomic and proteomic comparisons with paired normal gastric fibroblasts, our study suggests that TINAGL1 is a protein secreted from CAFs in DGC. TINAGL1 interacts with integrin β1 located on the membrane of cancer cells and activates the FAK signaling pathway to contribute to the progression of DGC. Finally, we found that the accumulation of TINAGL1^+^ CAFs in human DGCs tissues was significantly correlated with poor patient prognosis.

TINAGL1 was first identified in adrenocortical cells of mice [21]. TINAGL1 has been suggested to be a matricellular protein since it has been shown that TINAGL1 accumulates in the extracellular matrix and regulates cell function through interactions with several subtypes of integrins [22]. The interaction between TINAGL1 and integrin occurs during the post-implantation period of pregnancy [33]. However, its role in malignant tumors remains controversial. Public transcriptome data of patients with triple-negative breast cancer indicated that up-regulation of TINAGL1 was significantly correlated with better prognosis. TINAGL1 was defined as a competitive inhibitor for fibronectin-induced activation of FAK or EGFR, and consequently suppressed tumor progression in mouse models with triple-negative breast cancer [25]. TINAGL1 has been suggested as a potential therapeutic target to suppress metastasis in liver, lung, and gastric cancers [23, 24, 26]. In particular, a recent study suggested that TINAGL1 increases expression of MMPs in gastric cancer, and promotes the proliferation and migration of gastric cancer cells [26]. Most previous studies have focused on the expression of TINAGL1 in cancer cells themselves or could not consider the expression of host cells within tumors. Recent research has shown that fibroblasts could be a source of TINAGL1 during wound healing [27, 28]. Indeed, our multi-omics data suggest TINAGL1 as a specific marker of CAFs, and dual RNA in situ hybridization validated that TINAGL1 co-localizes with COL1A1, a fibroblast marker in human DGC tissues. Moreover, co-expression of TINAGL1 and COL1A1 in human DGC tissues significantly correlated with poor prognosis. Our experimental data, where inhibition of TINAGL1 in CAFs suppressed the progression of DGC, supported the clinical data. These results imply that CAF-derived TINAGL1 plays a critical role in DGC progression and could be a therapeutic target.

We identified FAK as an essential protein in the downstream signaling pathways associated with TINAGL1 and integrin β1 in DGC cells. FAK is a non-receptor tyrosine kinase that is considered a significant component of integrin-mediated signaling pathways [34, 35]. Several reports have shown that FAK activation can lead to up-regulation of EMT markers, such as Snail and Twist, through the PI3K/AKT and MAPK signaling pathways [36–38]. Thus, FAK has recently emerged as a potential target in cancer therapy [39, 40]. Some extracellular proteins, including CCN3 and ECM1, have been suggested as stimulators of integrin/FAK for invasion and metastasis of cancer cells. However, this depends on the type of cancer [36, 41]. Moreover, most studies did not propose a source of activators for the integrin/FAK pathways. Only one study reported that CAFs could activate FAK signaling in gastric cancer cells through the production of the extracellular protein lumican [42]. In the present study, we confirmed that CAF-induced FAK activation occurs and, through a multi-omics approach, found that TINAGL1 is a significant extracellular protein that leads to FAK activation in DGC cells. Moreover, TINAGL1 enhanced fibronectin induced FAK activation. According to a previous report [22], TINAGL1 binds to fibronectin and increase the total concentrations of integrin ligands. Consequently, interaction of TINAGL1 and fibronectin induces accumulation of ligand-activated integrins on plasma membrane in DGC cells. Several early phase clinical trials have investigated the efficacy of FAK inhibitors for treatment of advanced solid tumors, and found that humans could safely be administered FAK inhibitors [43–45]. However, this method has not been applied to gastric cancer. Our results suggest that the efficacy of FAK inhibitors should be investigated in patients with DGC in a clinical setting, and TINAGL1 expression in CAFs could be a diagnostic marker for the efficacy of FAK inhibitors. Based on the present study, integrin could be another CAF-specific target to inhibit the progression of DGC. In a clinical trial, anti-α4β7 integrin has shown efficacy in remission and clinical improvement for patients with inflammatory bowel disease [46, 47]. In addition, the small molecule inhibitor for integrin ανβ6 has been tested as an inhibitor of idiopathic pulmonary fibrosis [48]. Although it has not been used for cancer treatment in clinical settings, this clinically available drug could be applied to inhibit CAF-induced progression of DGC in the future.

In the present study, we performed dual RNA in-situ hybridization for TINAGL1 and COL1A1, a fibroblast marker, to identify CAF-specific expression of TINAGL1 in tissues of patients with DGC. Here, the accumulation of TINAGL1^+^ CAFs in DGC was significantly higher than that in paired normal gastric tissues and was associated with poor prognosis of DGC patients. However, we also found that CAFs without up-regulation of TINAGL1 existed, even in cancer tissues. Recent evidence has revealed CAF heterogeneity in solid tumors; therefore, molecular differences in CAF subtypes could indicate differences in function with respect to cancer progression [49, 50]. Single-cell RNA-seq (scRNA-seq) data for pancreatic ductal carcinoma indicated two major subtypes: inflammatory and myofibroblastic CAFs [51]. Our previous scRNA-seq data on DGC revealed that the fibroblasts within tumors could be subclassified into five subtypes, and one of the subtypes was relevant to the gene signature of myCAFs, which mainly upregulate extracellular matrix-related genes [52]. Interestingly, TINAGL1 was the top-ranked gene in the myCAF-like subtype in our scRNA-seq data for DGC. Thus, we suggest that TINAGL1 is a specific marker for one subtype of CAFs in DGC. As the role of myCAFs in tumor progression is still not clearly understood, the correlation between TINAGL1 and CAF subtypes should be investigated in future studies.

The present study had some limitations in proving the role of CAF-derived TINAGL1 in DGC. First, we did not determine the reason for the difference in expression between CAFs and NFs. A previous study suggested that tumor-derived factors such as TGFβ could convert normal quiescent fibroblasts into CAFs in gastric cancer [53]. Indeed, we confirmed that TGFβ could not up-regulate TINAGL1 in NFs (data not shown), and the mechanism of TINAGL1 up-regulation in CAFs should be investigated in the future. Second, we assessed molecular or functional differences between CAFs and NFs across multiple pairs due to the limited ability of primary cultured fibroblasts to maintain their characteristics over an extended period of time. Consequently, employing distinct fibroblast pairs for diverse experimental models was inevitable in this study. Third, we could not demonstrate the exclusive impact of recombinant TINAGL1 on the metastatic behavior of DGC cells (data not shown), despite the notable effectiveness observed with respect to genetic or pharmacological inhibition. Given the diversity of secretory factors derived from CAFs that intricately influence cancer cell progression, the influence of TINAGL1 alone may be insufficient to delineate comprehensive effects. Nevertheless, our findings suggest a pivotal role of CAF-secreted TINAGL1 in mediating the interplay between CAFs and cancer cells. Forth, studies have proposed a role of CAFs in tumor immunity [54, 55]. The experimental models in the present study were unable to reflect the interaction between CAFs and immune cells. We recently reported a syngeneic mouse model for DGC; thus, they could be useful in further studies [56].

## Conclusions

**Fig. 6:**
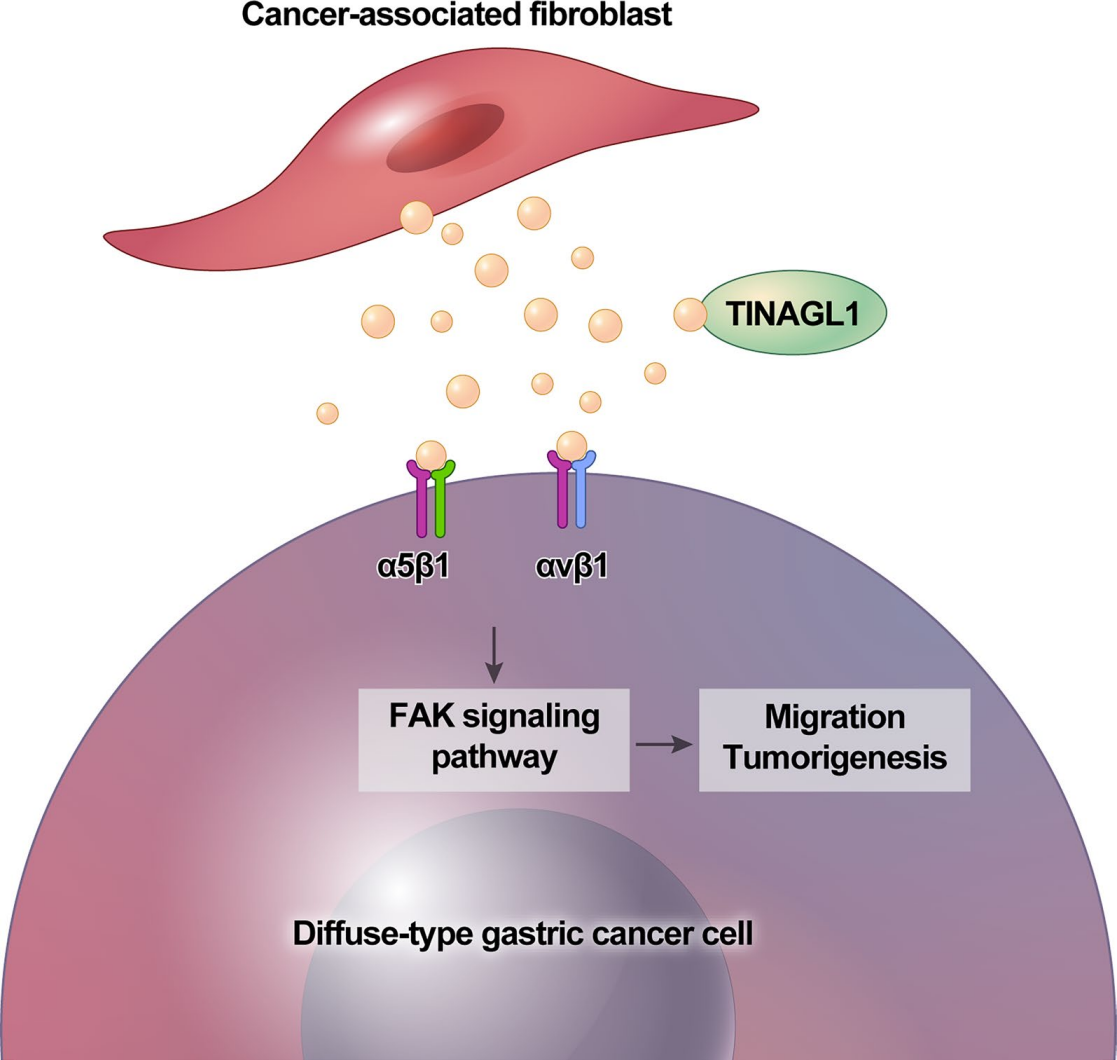
Summary of this study; CAF-secreted TINAGL1 binds to integrins on the DGC cancer cell membrane and promotes tumor progression through the FAK signaling pathway

In conclusion, our findings propose TINAGL1 as a distinctive secreted protein from CAFs in DGC. TINAGL1 appears to increase the aggressiveness of cancer cells by modulating the ITGB1/FAK signaling axis, as illustrated in Fig. 6. Notably, analysis of human specimens indicates an accumulation of TINAGL1^+^ CAFs in DGC tissues, establishing a significant correlation with tumor progression and oncologic outcomes. Based on our results, we advocate for considering TINAGL1^+^ CAFs as a potential biomarker for predicting the prognosis of DGC patients. Our findings suggest that interventions aimed at disrupting the TINAGL1/ITGB1/FAK axis between CAFs and cancer cells could hold therapeutic value in managing DGC patients.

### Supplementary Information

**Additional file 1: Fig. S1.** Schematic diagram for proteomic and transcriptome analysis. **Fig. S2.** (A) Western blotting for FAK phosphorylation in fibroblast co-cultured MKN45 and KATO-III cells. Representative images and graph of the transwell migration assay of MKN45 (B) and KATO-III (C) with or without fibroblasts (magnification, × 100). **Fig. S3.** Normalized protein expression by housekeeping genes from Fig. 1A (A), 1E (B), 1F (C), 1G (D), 4B (E), and 4E (F). **Fig. S4.** (A) Cell proliferation assay for NF- or CAF-CM treated SNU601 cells. (B) qRT-PCR for TINAGL1 gene expression in NF-CAF133 pair. Tumor volume (C) and weight (D) for Fig. 3E. (E) Representative images for H&E and immunohistochemistry staining at the tumor margin (scale bars, 50 μm). **Fig. S5.** (A) RT-PCR for *hTERT* and *TINAGL1* expression in wild-type and immortalized NF-CAF47 pairs. (C) Representative images and graph of the transwell migration assay of immortalized fibroblast co-cultured SNU601 cells (magnification, × 100). Data were analyzed using the Kruskal–Wallis test with Dunn’s test. *P < 0.05. **Fig. S6.** (A) Western blotting for Twist expression in siRNA transfected CAF47 co-cultured SNU601 cells. (B) Western blotting for EMT marker expression in PF-573,228-treated SNU601 cells. Tumor weight (C) and volume graph (D) for Fig. 4I. (E) Representative images for H&E and immunohistochemistry staining at the tumor margin (scale bars, 50 μm). **Fig. S7.** Gene expression correlation between *TINAGL1* and *ITGB1*, *ITGA5*, and *ITGAV* from the GSE15459 (A, n = 200) and TCGA-STAD datasets (B, n = 375). **Fig. S8.** (A) Kaplan–Meier plots for *TINAGL1* and *COL1A1*, *ACTA2*, or *FAP* expression in intestinal-type gastric cancer patients from the GSE15459 dataset (n = 99). (B) Kaplan–Meier plots for *TINAGL1* and *COL1A1*, *ACTA2*, or *FAP* expression in gastric cancer patients from the TCGA-STAD dataset (n = 54 for diffuse, 155 for intestinal).

**Additional file 2: Table S1.** Clinical characteristics of three diffuse-type gastric cancer patients involved in RNA sequencing.

**Additional file 3: Table S2.** Primer sequences for RT-PCR.

**Additional file 4: Table S3.** Primer sequences for qRT-PCR.

**Additional file 5: Table S4.** Eight up-regulated molecules in CAF16.

**Additional file 6: Table S5.** Fifteen up-regulated molecules in CAF20.

**Additional file 7: Table S6.** *TINAGL1* and *ACTA2* expression is associated with overall survival of DGC patients in the GSE15459 dataset.

**Additional file 8: Table S7.** *TINAGL1* and *FAP* expression is associated with overall survival of DGC patients in the GSE15459 dataset.

## Data Availability

The datasets generated or analyzed in the current study are available in the GEO database (GSE233331 and GSE15459) and TCGA Research Network (https://www.cancer.gov/tcga).

## Abbreviations

DGC: Diffuse-type gastric cancer
CAF: Cancer-associated fibroblast
NF: Normal fibroblast
CM: Conditioned-media
TINAGL1: Tubulointerstitial nephritis antigen-like 1
FFPE: Formalin-fixed, paraffin-embedded
RNA-ISH: RNA-in situ hybridization
FAK: Focal adhesion kinase
COL1A1: Collagen type 1 alpha 1
GC: Gastric cancer
TME: Tumor microenvironment
AZ-1: Adrenocortical zonation factor-1
LCN7: Lipocalin-7
ECM: Extracellular matrix
MMP: Matrix metalloproteinase
DEG: Differentially expressed gene
RT-PCR: Reverse transcription polymerase chain reaction
qRT-PCR: Quantitative reverse transcription polymerase chain reaction
ELISA: Enzyme-linked immunosorbent assay
IP: Immunoprecipitation
siRNA: Small interfering RNA
shRNA: Short hairpin RNA
IHC: Immunohistochemistry
TCGA-STAD: The Cancer Genome Atlas Stomach Adenocarcinoma
SE: Standard error
GSEA: Gene set enrichment analysis
LC–MS/MS: Liquid chromatography-tandem mass spectrometry
ACTA2: Actin alpha 2 smooth muscle
FAP: Fibroblast activation protein alpha
HR: Hazard ratio
CI: Confidence interval
