# Supplementary material for: Tubulointerstitial nephritis antigen-like 1 from cancer-associated fibroblasts contribute to the progression of diffuse-type gastric cancers through the interaction with integrin β1
Source: J Transl Med. 2024 Feb 14;22:154. doi: 10.1186/s12967-024-04963-9 (PMC10868052; doi:10.1186/s12967-024-04963-9)
Supplement: Supplementary file 1 — Additional file 1: Fig. S1. Schematic diagram for proteomic and transcriptome analysis. Fig. S2. (A) Western blotting for FAK phosphorylation in fibroblast co-cultured MKN45 and KATO-III cells. Representative images and graph of the transwell migration assay of MKN45 (B) and KATO-III (C) with or without fibroblasts (magnification, × 100). Fig. S3. Normalized protein expression by housekeeping genes from Fig. 1A (A), 1E (B), 1F (C), 1G (D), 4B (E), and 4E (F). Fig. S4. (A) Cell proliferation assay for NF- or CAF-CM treated SNU601 cells. (B) qRT-PCR for TINAGL1 gene expression in NF-CAF133 pair. Tumor volume (C) and weight (D) for Fig. 3E. (E) Representative images for H&E and immunohistochemistry staining at the tumor margin (scale bars, 50 μm). Fig. S5. (A) RT-PCR for hTERT and TINAGL1 expression in wild-type and immortalized NF-CAF47 pairs. (C) Representative images and graph of the transwell migration assay of immortalized fibroblast co-cultured SNU601 cells (magnification, × 100). Data were analyzed using the Kruskal–Wallis test with Dunn’s test. *P < 0.05. Fig. S6. (A) Western blotting for Twist expression in siRNA transfected CAF47 co-cultured SNU601 cells. (B) Western blotting for EMT marker expression in PF-573,228-treated SNU601 cells. Tumor weight (C) and volume graph (D) for Fig. 4I. (E) Representative images for H&E and immunohistochemistry staining at the tumor margin (scale bars, 50 μm). Fig. S7. Gene expression correlation between TINAGL1 and ITGB1, ITGA5, and ITGAV from the GSE15459 (A, n = 200) and TCGA-STAD datasets (B, n = 375). Fig. S8. (A) Kaplan–Meier plots for TINAGL1 and COL1A1, ACTA2, or FAP expression in intestinal-type gastric cancer patients from the GSE15459 dataset (n = 99). (B) Kaplan–Meier plots for TINAGL1 and COL1A1, ACTA2, or FAP expression in gastric cancer patients from the TCGA-STAD dataset (n = 54 for diffuse, 155 for intestinal). [file 12967_2024_4963_MOESM1_ESM.docx]

**
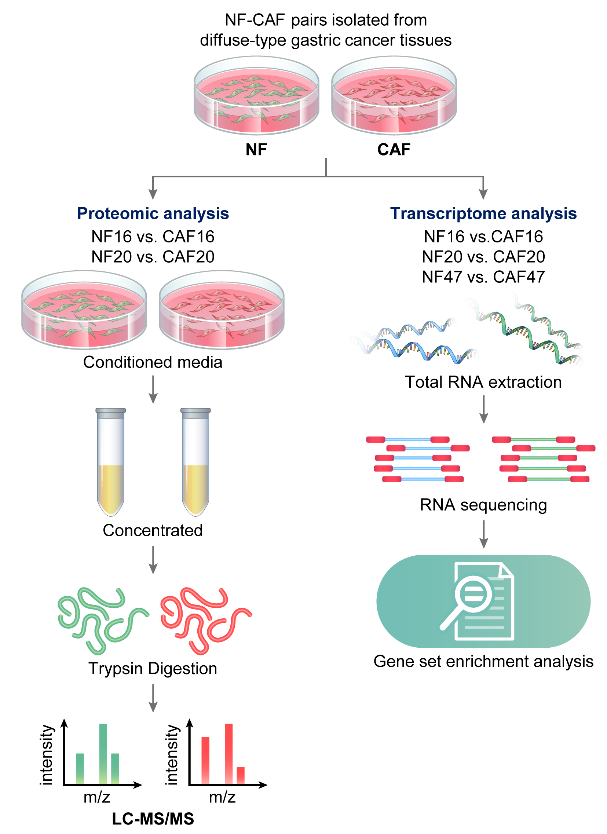
**

**Fig. S1** Schematic diagram for proteomic and transcriptome analysis.

**
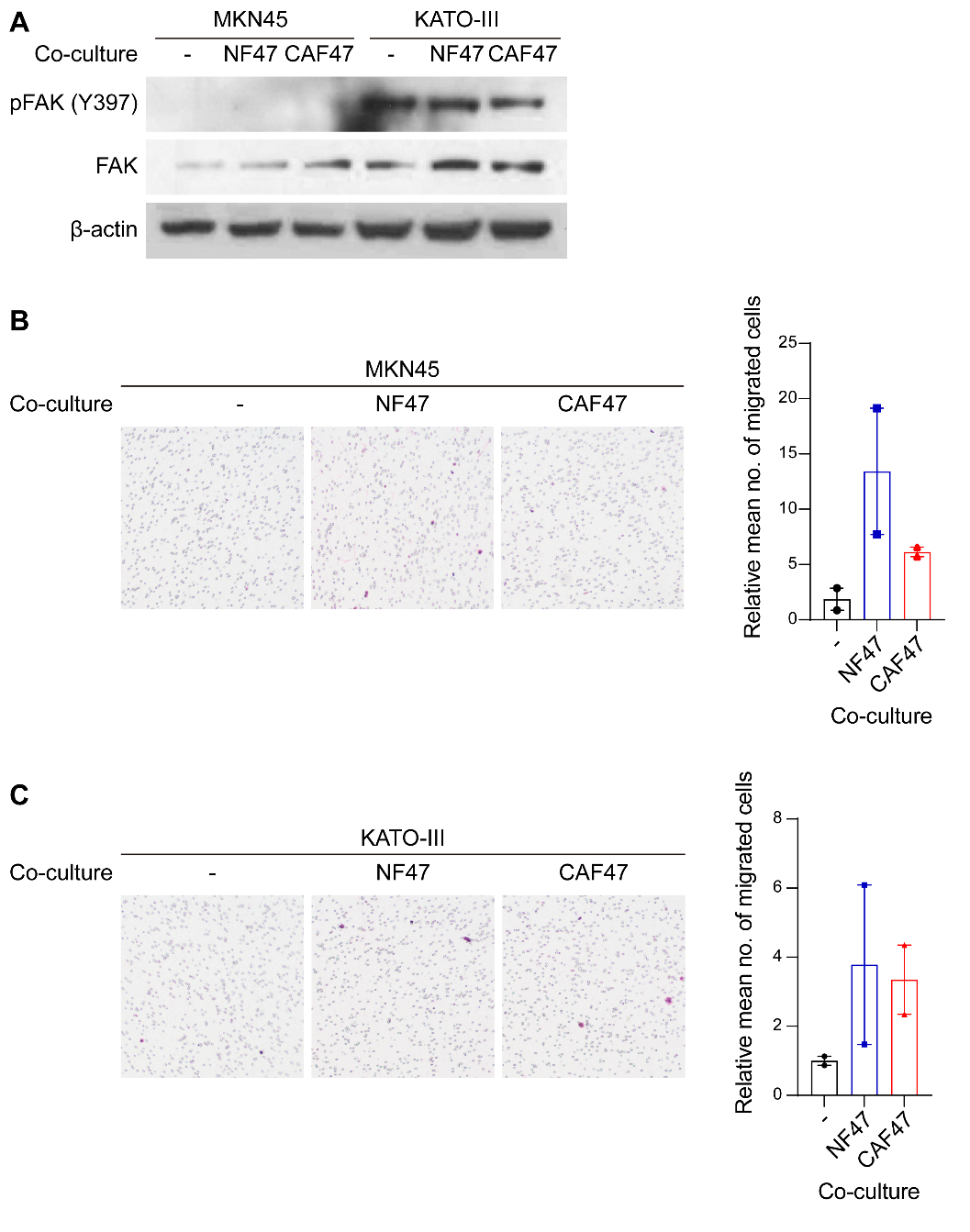
**

**Fig. S2** (A) Western blotting for FAK phosphorylation in fibroblast co-cultured MKN45 and KATO-III cells. Representative images and graph of the transwell migration assay of MKN45 (B) and KATO-III (C) with or without fibroblasts (magnification, x100).

**
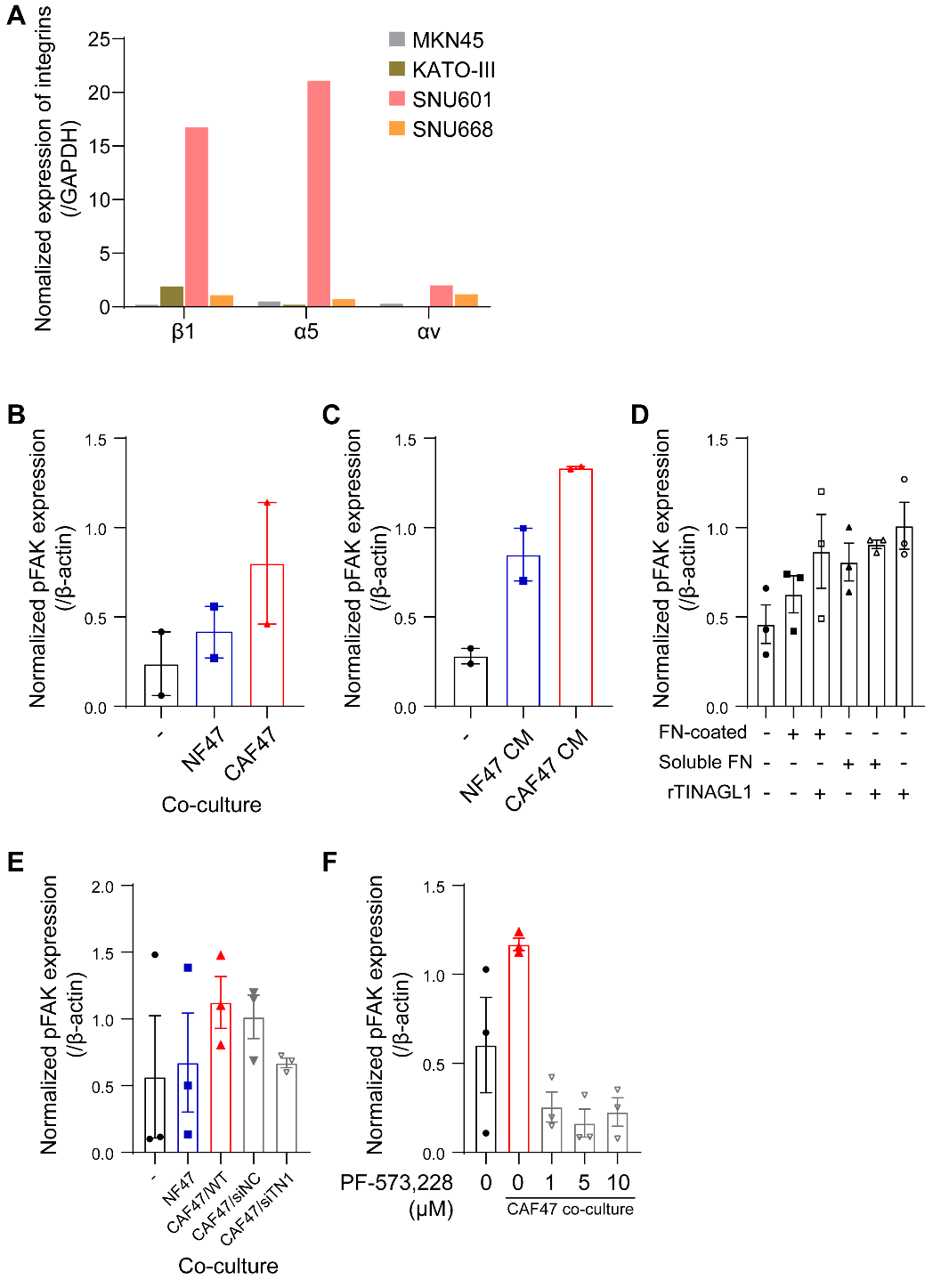
**

**Fig. S3** Normalized protein expression by housekeeping genes from Fig. 1A (A), 1E (B), 1F (C), 1G (D), 4B (E), and 4E (F).

**
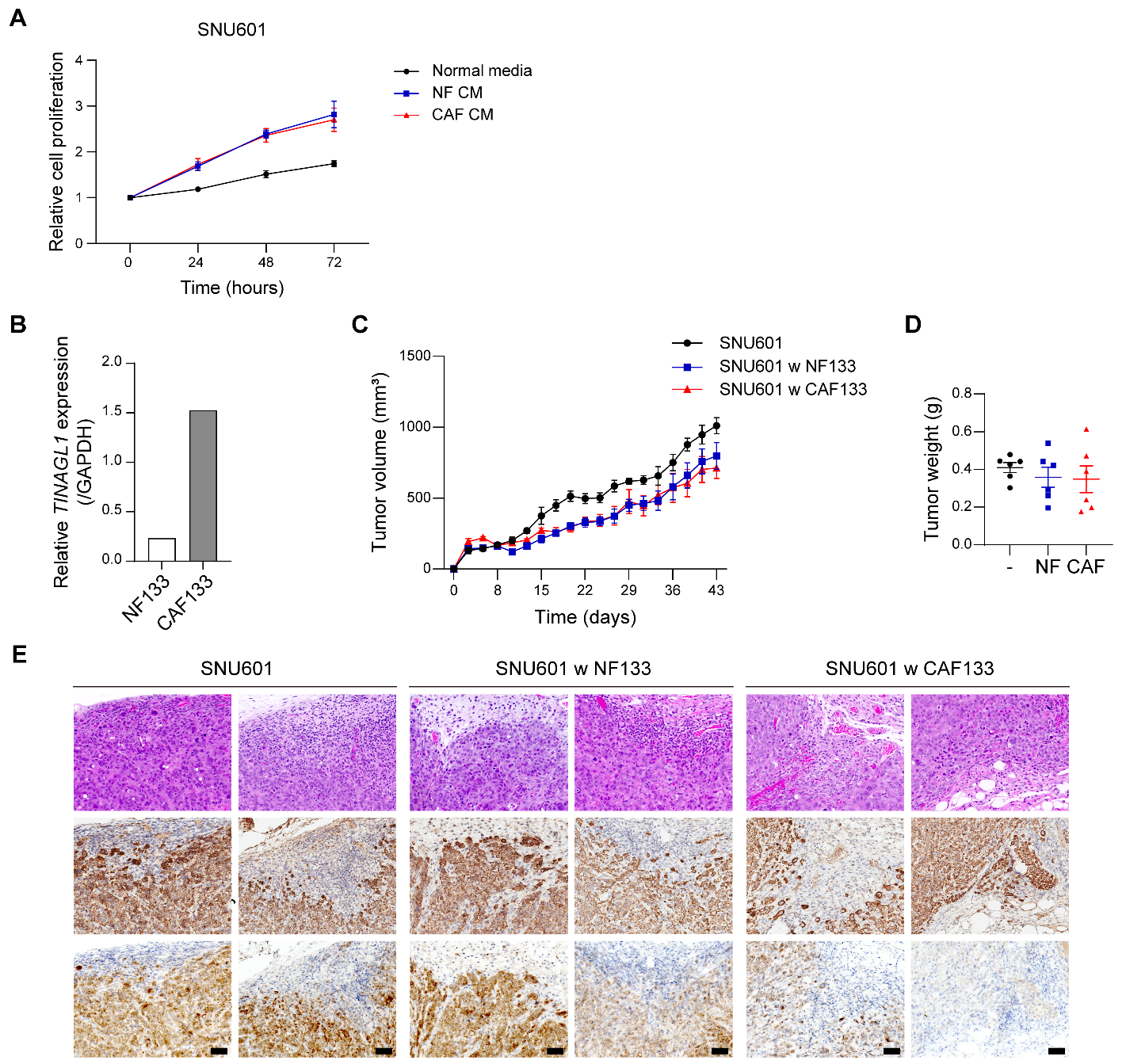
**

**Fig. S4** (A) Cell proliferation assay for NF- or CAF-CM treated SNU601 cells. (B) qRT-PCR for TINAGL1 gene expression in NF-CAF133 pair. Tumor volume (C) and weight (D) for Fig. 3E. (E) Representative images for H&E and immunohistochemistry staining at the tumor margin (scale bars, 50 μm).

**
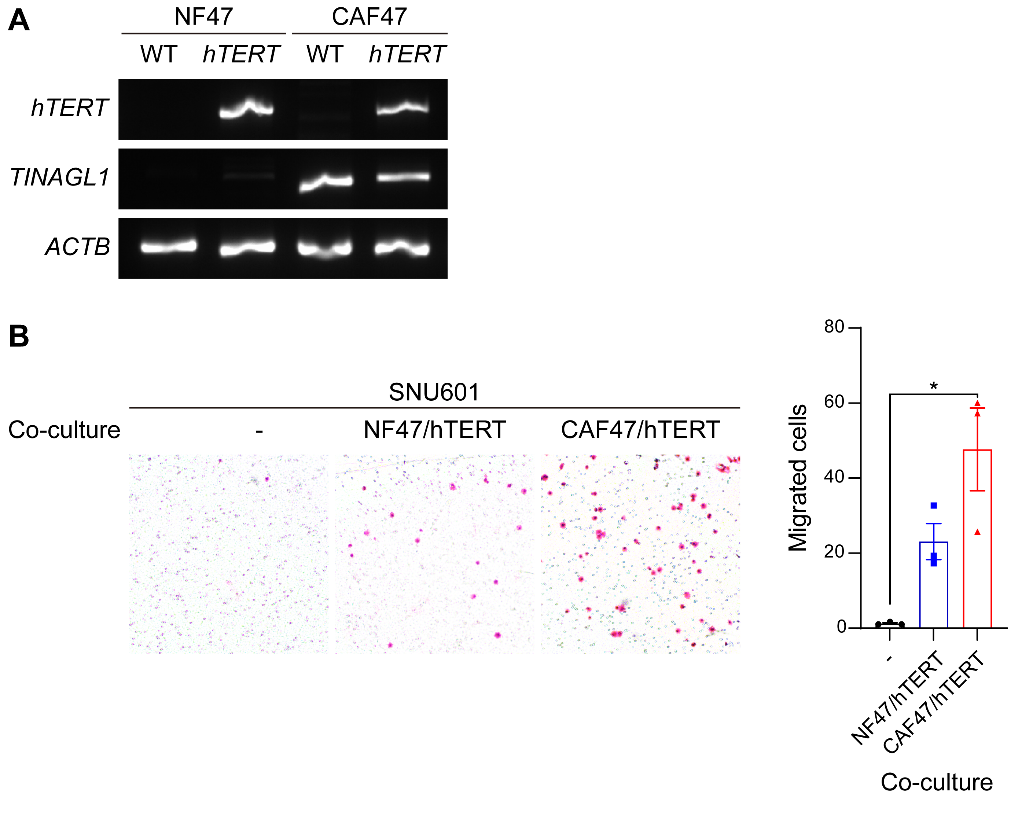
**

**Fig. S5** (A) RT-PCR for *hTERT* and *TINAGL1* expression in wild-type and immortalized NF-CAF47 pairs. (C) Representative images and graph of the transwell migration assay of immortalized fibroblast co-cultured SNU601 cells (magnification, x100). Data were analyzed using the Kruskal-Wallis test with Dunn’s test. **P* < 0.05.

**
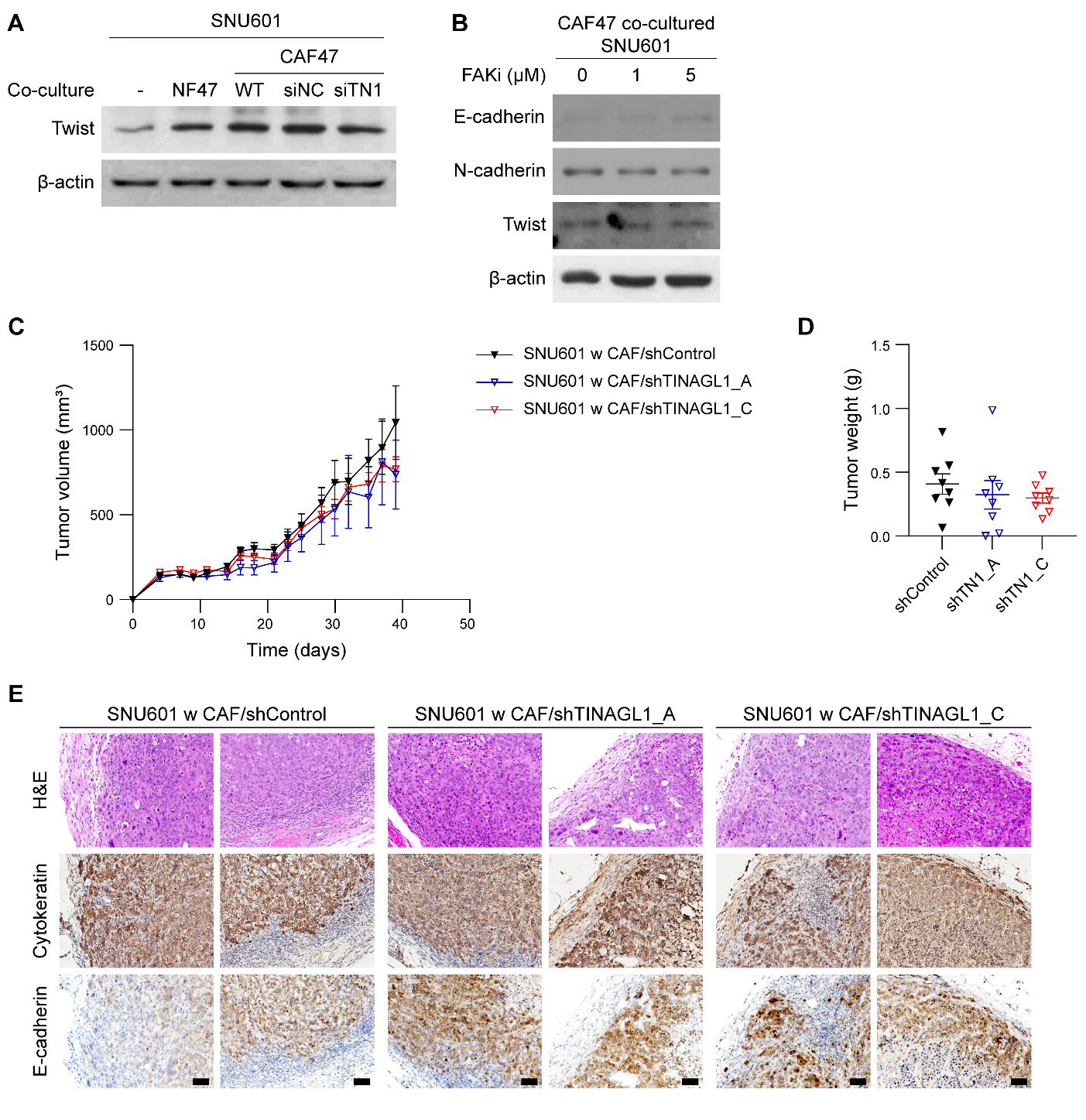
**

**Fig. S6** (A) Western blotting for Twist expression in siRNA transfected CAF47 co-cultured SNU601 cells. (B) Western blotting for EMT marker expression in PF-573,228-treated SNU601 cells. Tumor weight (C) and volume graph (D) for Fig. 4I. (E) Representative images for H&E and immunohistochemistry staining at the tumor margin (scale bars, 50 μm).


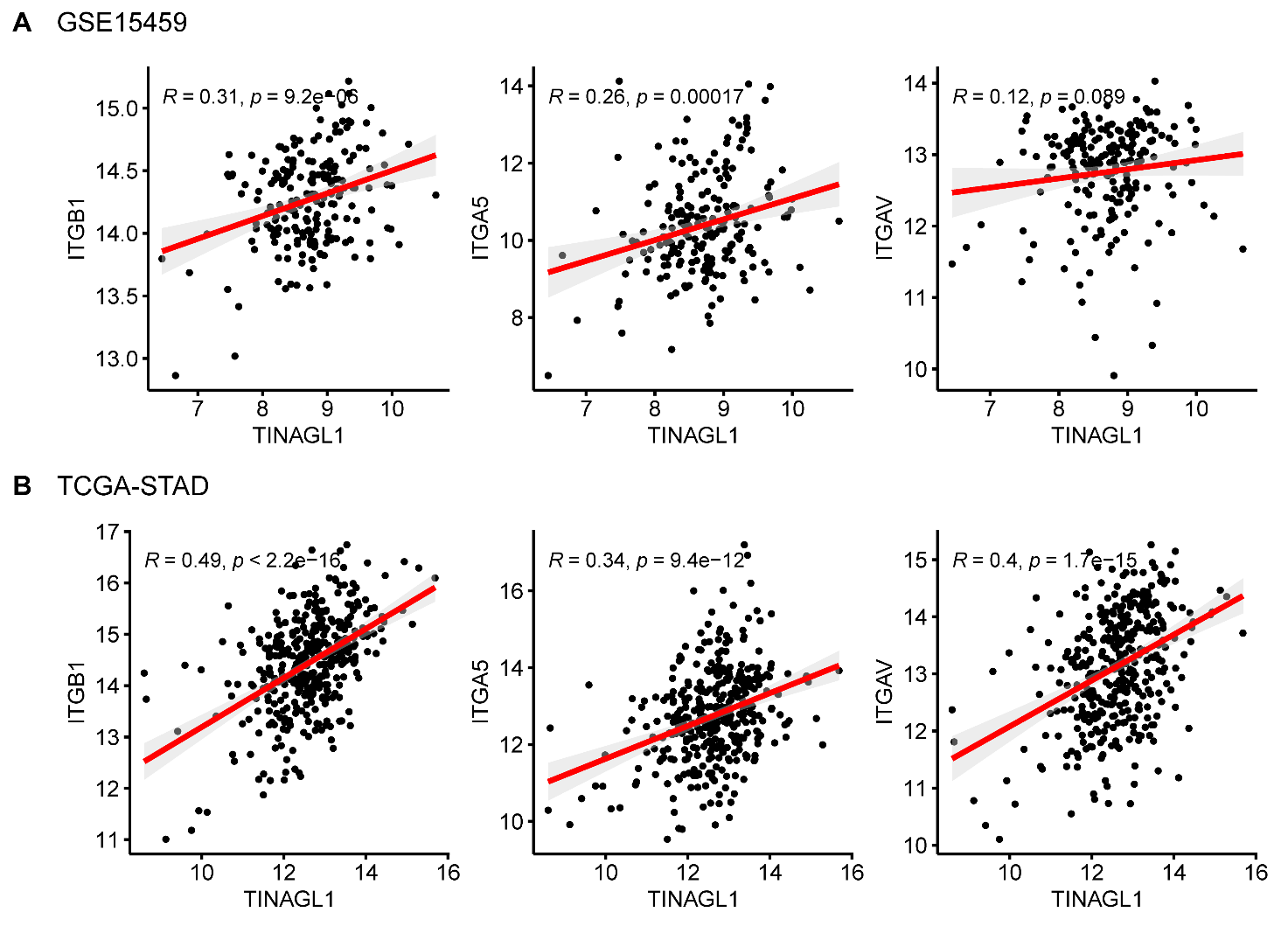


**Fig. S7** Gene expression correlation between *TINAGL1* and *ITGB1*, *ITGA5*, and *ITGAV* from GSE15459 (A, n = 200) and TCGA-STAD datasets (B, n = 375).

**
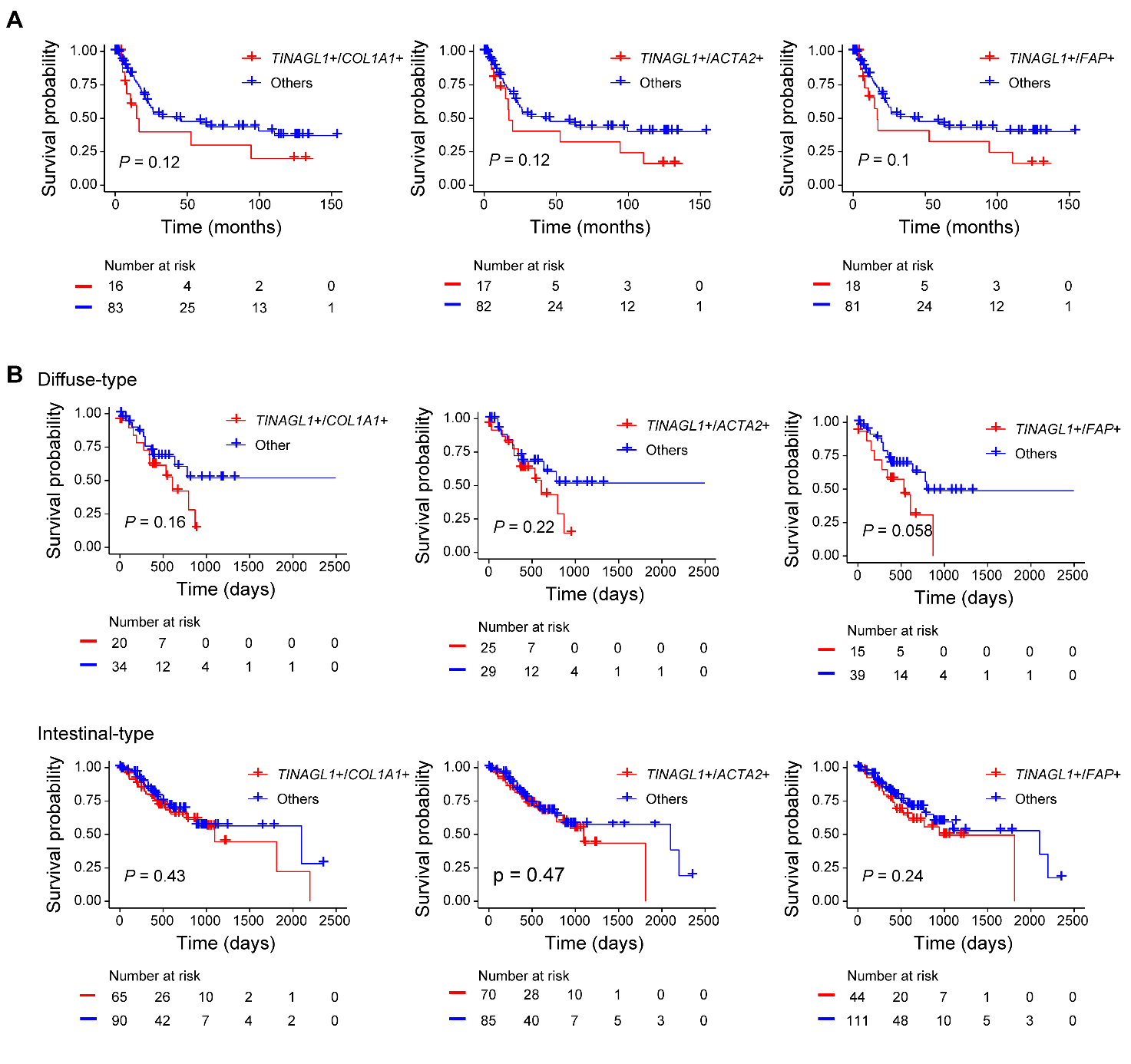
**

**Fig. S8** (A) Kaplan-Meier plots for *TINAGL1* and *COL1A1*, *ACTA2*, or *FAP* expression in intestinal-type gastric cancer patients from the GSE15459 dataset (n = 99). (B) Kaplan-Meier plots for *TINAGL1* and *COL1A1*, *ACTA2*, or *FAP* expression in gastric cancer patients from the TCGA-STAD dataset (n = 54 for diffuse, 155 for intestinal).
