# Supplementary material for: Tubulointerstitial nephritis antigen-like 1 from cancer-associated fibroblasts contribute to the progression of diffuse-type gastric cancers through the interaction with integrin β1
Source: J Transl Med. 2024 Feb 14;22:154. doi: 10.1186/s12967-024-04963-9 (PMC10868052; doi:10.1186/s12967-024-04963-9)
Supplement: Supplementary file 2 — Additional file 2: Table S1. Clinical characteristics of three diffuse-type gastric cancer patients involved in RNA sequencing. [file 12967_2024_4963_MOESM2_ESM.docx]

**Table S1.** Clinical characteristics of three diffuse-type gastric cancer patients involved in RNA sequencing.

| **Patient No.** | **Age** | **Gender** | **T** | **N** | **M** | **Stage** | **Max tumor size (cm)** | **Min tumor size (cm)** | **WHO classification** |
| --- | --- | --- | --- | --- | --- | --- | --- | --- | --- |
| 16 | 55 | Male | T4b | N3b | M0 | IIIC | 15 | 12 | Signet ring cell carcinoma |
| 20 | 57 | Male | T3 | N3a | M0 | IIIB | 6 | 5 | Signet ring cell carcinoma |
| 47 | 61 | Male | T3 | N3a | M0 | IIIB | 12 | 6 | Signet ring cell carcinoma |
