# Supplementary material for: Tubulointerstitial nephritis antigen-like 1 from cancer-associated fibroblasts contribute to the progression of diffuse-type gastric cancers through the interaction with integrin β1
Source: J Transl Med. 2024 Feb 14;22:154. doi: 10.1186/s12967-024-04963-9 (PMC10868052; doi:10.1186/s12967-024-04963-9)
Supplement: Supplementary file 3 — Additional file 3: Table S2. Primer sequences for RT-PCR. [file 12967_2024_4963_MOESM3_ESM.docx]

**Table S2.** Primer sequences for RT-PCR.

| **Gene name** | **Sequence (5’-3’)** |
| --- | --- |
| *TINAGL1*_Forward | TGT GAC CTC TTC TGC AAC CG |
| *TINAGL1*_Reverse | CCC TGG GTT CAG CAC TGT ATA A |
| *hTERT*_Forward | CTG GAC GAT ATC CAC AGG GC |
| *hTERT*_Reverse | AAG TTC ACC ACG CAG CCA TA |
| *ACTB*_Forward | TCA AGA TCA TTG CTC CTC CTG AGC |
| *ACTB*_Reverse | TGC TGT CAC CTT CAC CGT TCC AGT |
