# Supplementary material for: Tubulointerstitial nephritis antigen-like 1 from cancer-associated fibroblasts contribute to the progression of diffuse-type gastric cancers through the interaction with integrin β1
Source: J Transl Med. 2024 Feb 14;22:154. doi: 10.1186/s12967-024-04963-9 (PMC10868052; doi:10.1186/s12967-024-04963-9)
Supplement: Supplementary file 4 — Additional file 4: Table S3. Primer sequences for qRT-PCR. [file 12967_2024_4963_MOESM4_ESM.docx]

**Table S3.** Primer sequences for qRT-PCR.

| **Gene name** | **Sequence (5’-3’)** |
| --- | --- |
| *TINAGL1*_Forward | CTA CCT GGG CGC CAT CTG TT |
| *TINAGL1*_Reverse | CAT ACA TCC TTG GAT CGG GG |
| *GAPDH*_Forward | GAG TCA ACG GAT TTG GTC GT |
| *GAPDH*_Reverse | TGG AAG ATG GTG ATG GGA TT |
