# Supplementary material for: Tubulointerstitial nephritis antigen-like 1 from cancer-associated fibroblasts contribute to the progression of diffuse-type gastric cancers through the interaction with integrin β1
Source: J Transl Med. 2024 Feb 14;22:154. doi: 10.1186/s12967-024-04963-9 (PMC10868052; doi:10.1186/s12967-024-04963-9)
Supplement: Supplementary file 5 — Additional file 5: Table S4. Eight up-regulated molecules in CAF16. [file 12967_2024_4963_MOESM5_ESM.docx]

**Table S4.** Eight up-regulated molecules in CAF16.

|  | **CAF16/NF16 log2FC^†^** | |
| --- | --- | --- |
| **Name** | **Gene** | **Protein** |
| *SFRP4* | 1.019 | 1.838 |
| *TINAGL1* | 4.864 | 22.543 |
| *SRGN* | 1.945 | 6.602 |
| *BGN* | 1.363 | 26.520 |
| *CDH13* | 1.527 | 2.421 |
| *FMOD* | 1.056 | 22.993 |
| *A2M* | 3.227 | 24.325 |
| *CPE* | 1.959 | 3.910 |
| FC**^†^**, fold-change. | | |
