# Supplementary material for: Tubulointerstitial nephritis antigen-like 1 from cancer-associated fibroblasts contribute to the progression of diffuse-type gastric cancers through the interaction with integrin β1
Source: J Transl Med. 2024 Feb 14;22:154. doi: 10.1186/s12967-024-04963-9 (PMC10868052; doi:10.1186/s12967-024-04963-9)
Supplement: Supplementary file 6 — Additional file 6: Table S5. Fifteen up-regulated molecules in CAF20. [file 12967_2024_4963_MOESM6_ESM.docx]

**Table S5.** Fifteen up-regulated molecules in CAF20.

|  | CAF20/NF20 log2FC^†^ | |
| --- | --- | --- |
| **Name** | Gene | Protein |
| *CHI3L1* | 4.544 | 20.579 |
| *TINAGL1* | 2.809 | 6.150 |
| *CHRDL2* | 2.596 | 21.472 |
| *PODN* | 1.755 | 22.755 |
| *A2M* | 3.152 | 26.924 |
| *CLU* | 1.313 | 4.013 |
| *ADAMTS8* | 2.375 | 24.339 |
| *MFAP5* | 2.570 | 22.961 |
| *TNFRSF11B* | 1.300 | 21.658 |
| *LAMA4* | 1.032 | 3.457 |
| *FLNC* | 1.358 | 2.312 |
| *THY1* | 1.244 | 25.932 |
| *CCL2* | 2.603 | 2.588 |
| *LAMA5* | 2.695 | 5.594 |
| *GLUL* | 1.739 | 21.904 |
| FC**^†^**, fold-change. | | |
