# Supplementary material for: Tubulointerstitial nephritis antigen-like 1 from cancer-associated fibroblasts contribute to the progression of diffuse-type gastric cancers through the interaction with integrin β1
Source: J Transl Med. 2024 Feb 14;22:154. doi: 10.1186/s12967-024-04963-9 (PMC10868052; doi:10.1186/s12967-024-04963-9)
Supplement: Supplementary file 7 — Additional file 7: Table S6. TINAGL1 and ACTA2 expression is associated with overall survival of DGC patients in the GSE15459 dataset. [file 12967_2024_4963_MOESM7_ESM.docx]

**Table S6.** *TINAGL1* and *ACTA2* expression is associated with overall survival of DGC patients in the GSE15459 dataset.

|  |  |  | **Univariate** | |  | **Multivariate** | |
| --- | --- | --- | --- | --- | --- | --- | --- |
| **Variable** | | **n (%)** | **HR^†^ (95% CI^‡^)** | ***P*-Value** |  | **HR (95% CI)** | ***P*-Value** |
| *TINAGL1/ACTA2* | |  |  |  |  |  |  |
|  | Others | 61 (81.3) | - |  |  | - |  |
|  | *TINAGL1*+/*ACTA2*+ | 14 (18.7) | 2.13 (1.04-4.39) | 0.040 |  | 2.81 (1.18-6.71) | 0.020 |
| Age | |  |  |  |  |  |  |
|  | >65 | 31 (41.3) | - |  |  | - |  |
|  | 65≥ | 44 (58.7) | 0.80 (0.43-1.51) | 0.498 |  | 0.75 (0.35-1.62) | 0.470 |
| Gender | |  |  |  |  |  |  |
|  | Female | 39 (52.0) | - |  |  | - |  |
|  | Male | 36 (48.0) | 1.55 (0.83-2.90) | 0.170 |  | 1.01 (0.49-2.10) | 0.977 |
| Stage | |  |  |  |  |  |  |
|  | I | 9 (12.0) | - |  |  | - |  |
|  | II | 12 (16.0) | 7.98 (0.98-65.10) | 0.052 |  | 8.44 (0.99-71.71) | 0.051 |
|  | III | 31 (41.3) | 9.49 (1.25-72.24) | 0.030 |  | 11.48 (1.42-92.96) | 0.022 |
|  | IV | 23 (30.7) | 33.96 (4.36-264.71) | 0.001 |  | 50.99 (5.99-434.29) | <0.001 |
| Subtype | |  |  |  |  |  |  |
|  | Metabolic | 16 (26.2) | - |  |  | - |  |
|  | Proliferative | 12 (19.7) | 1.51 (0.54-4.20) | 0.427 |  | 0.81 (0.28-2.30) | 0.689 |
|  | Mesenchymal | 33 (54.1) | 1.60 (0.71-3.63) | 0.256 |  | 1.04 (0.44-2.49) | 0.928 |

Cox regression model; HR**^†^**, hazard ratio; CI**^‡^**, confidence interval
