# Supplementary material for: Tubulointerstitial nephritis antigen-like 1 from cancer-associated fibroblasts contribute to the progression of diffuse-type gastric cancers through the interaction with integrin β1
Source: J Transl Med. 2024 Feb 14;22:154. doi: 10.1186/s12967-024-04963-9 (PMC10868052; doi:10.1186/s12967-024-04963-9)
Supplement: Supplementary file 8 — Additional file 8: Table S7. TINAGL1 and FAP expression is associated with overall survival of DGC patients in the GSE15459 dataset. [file 12967_2024_4963_MOESM8_ESM.docx]

**Table S7.** *TINAGL1* and *FAP* expression is associated with overall survival of DGC patients in the GSE15459 dataset.

|  |  |  | **Univariate** | |  | **Multivariate** | |
| --- | --- | --- | --- | --- | --- | --- | --- |
| **Variable** | | **n (%)** | **HR^†^ (95% CI^‡^)** | ***P*-Value** |  | **HR (95% CI)** | ***P*-Value** |
| *TINAGL1/FAP* | |  |  |  |  |  |  |
|  | Others | 64 (85.3) | - |  |  | - |  |
|  | *TINAGL1*+/*FAP*+ | 11 (14.7) | 4.21 (2.00-8.88) | <0.001 |  | 4.39 (1.78-10.82) | 0.001 |
| Age | |  |  |  |  |  |  |
|  | >65 | 31 (41.3) | - |  |  | - |  |
|  | 65≥ | 44 (58.7) | 0.80 (0.43-1.51) | 0.498 |  | 0.70 (0.32-1.52) | 0.371 |
| Gender | |  |  |  |  |  |  |
|  | Female | 39 (52.0) | - |  |  | - |  |
|  | Male | 36 (48.0) | 1.55 (0.83-2.90) | 0.170 |  | 1.13 (0.55-2.36) | 0.735 |
| Stage | |  |  |  |  |  |  |
|  | I | 9 (12.0) | - |  |  | - |  |
|  | II | 12 (16.0) | 7.98 (0.98-65.10) | 0.052 |  | 6.01 (0.71-50.96) | 0.100 |
|  | III | 31 (41.3) | 9.49 (1.25-72.24) | 0.030 |  | 8.72 (1.07-71.19) | 0.043 |
|  | IV | 23 (30.7) | 33.96 (4.36-264.71) | 0.001 |  | 36.87 (4.44-306.34) | 0.001 |
| Subtype | |  |  |  |  |  |  |
|  | Metabolic | 16 (26.2) | - |  |  | - |  |
|  | Proliferative | 12 (19.7) | 1.51 (0.54-4.20) | 0.427 |  | 0.92 (0.32-2.61) | 0.871 |
|  | Mesenchymal | 33 (54.1) | 1.60 (0.71-3.63) | 0.256 |  | 1.05 (0.44-2.50) | 0.919 |

Cox regression model; HR**^†^**, hazard ratio; CI**^‡^**, confidence interval
